# Lipids, obesity and gallbladder disease in women: insights from genetic studies using the cardiovascular gene-centric 50K SNP array

**DOI:** 10.1038/ejhg.2015.63

**Published:** 2015-04-29

**Authors:** Santiago Rodriguez, Tom R Gaunt, Yiran Guo, Jie Zheng, Michael R Barnes, Weihang Tang, Fazal Danish, Andrew Johnson, Berta A Castillo, Yun R Li, Hakon Hakonarson, Sarah G Buxbaum, Tom Palmer, Michael Y Tsai, Leslie A Lange, Shah Ebrahim, George Davey Smith, Debbie A Lawlor, Aaron R Folsom, Ron Hoogeveen, Alex Reiner, Brendan Keating, Ian NM Day

**Affiliations:** 1 Bristol Genetic Epidemiology Laboratories, School of Social and Community Medicine, University of Bristol, Bristol, UK; 2 MRC Integrative Epidemiology Unit (IEU), School of Social and Community Medicine, University of Bristol, Bristol, UK; 3 Division of Transplantation, Department of Surgery, University of Pennsylvania, Philadelphia, PA, USA; 4 BGI-Shenzhen, Beishan Industrial Zone, Shenzhen, China; 5 William Harvey Research Institute National Institute for Health Biomedical Research Unit, Barts and the London School of Medicine, Queen Mary University of London, London, UK; 6 Division of Epidemiology and Community Health, University of Minnesota, Minneapolis, MN, USA; 7 National Heart, Lung and Blood Institute, The Framingham Heart Study, Framingham, MA, USA; 8 Jackson Heart Study, Jackson State University, Jackson, MS; School of Health Sciences, Department of Epidemiology and Biostatistics, Jackson State University, Jackson, MS, USA; 9 Division of Health Sciences, Warwick Medical School, University of Warwick, Coventry, UK; 10 Department of Laboratory Medicine and Pathology, University of Minnesota, Minneapolis, MN, USA; 11 Department of Genetics, University of North Carolina School of Medicine at Chapel Hill, Chapel Hill, NC, USA; 12 Department of Non-communicable Disease Epidemiology, The London School of Hygiene & Tropical Medicine, London, UK; 13 Department of Medicine, Baylor College of Medicine, Houston, TX, USA; 14 Division of Public Health Sciences, Fred Hutchinson Cancer Research Center, Seattle, WA, USA; 15 Department of Pediatrics, University of Pennsylvania, Philadelphia, PA, USA

## Abstract

Gallbladder disease (GBD) has an overall prevalence of 10–40% depending on factors such as age, gender, population, obesity and diabetes, and represents a major economic burden. Although gallstones are composed of cholesterol by-products and are associated with obesity, presumed causal pathways remain unproven, although BMI reduction is typically recommended. We performed genetic studies to discover candidate genes and define pathways involved in GBD. We genotyped 15 241 women of European ancestry from three cohorts, including 3216 with GBD, using the Human cardiovascular disease (HumanCVD) BeadChip containing up to ~53 000 single-nucleotide polymorphisms (SNPs). Effect sizes with *P*-values for development of GBD were generated. We identify two new loci associated with GBD, *GCKR* rs1260326:T>C (*P*=5.88 × 10^−7^, *ß*=−0.146) and *TTC39B* rs686030:C>A (*P*=6.95x10^−7^, *ß*=0.271) and detect four independent SNP effects in *ABCG8* rs4953023:G>A (*P*=7.41 × 10^−47^, *ß*=0.734), *ABCG8* rs4299376:G^>^T (*P*=2.40 × 10^−18^, *ß*=0.278), *ABCG5* rs6544718:T>C (*P*=2.08 × 10^−14^, *ß*=0.044) and *ABCG5* rs6720173:G>C (*P*=3.81 × 10^−12^, *ß*^=^0.262) in conditional analyses taking genotypes of rs4953023:G>A as a covariate. We also delineate the risk effects among many genotypes known to influence lipids. These data, from the largest GBD genetic study to date, show that specific, mainly hepatocyte-centred, components of lipid metabolism are important to GBD risk in women. We discuss the potential pharmaceutical implications of our findings.

## Introduction

Gallbladder disease (GBD) is a major cause of morbidity, hospital admission, surgical intervention and economic burden and is caused by gallstones.^1^ Gallstones occur in 10–40% of adults in developed countries,^2^ predominantly in women.^3^ The incidence increases with age and other factors such as obesity and diabetes, although there are differences between men and women in the determinants of GBD.^4^ These differences make advisable the analysis of the genetic basis of GBD separate by sex. Causes of morbidity include biliary colic, cholecystitis, choledocholithiasis and pancreatitis.^5^ In the USA, >700 000 cholecystectomies are undertaken per year.^6^ Large genetic studies have shown that the additive genetic heritability of symptomatic gallstones ranges from 25 to 29%.^7, 8^

In developed countries, most gallstones are formed of cholesterol, but pigment stones consisting substantially of calcium bilirubinate predominate in regions where bacterial and parasitic infections of the biliary tree, and hemolysis, are common.^9^ Exogenous estrogens are a risk factor, as are fibrates.^10^

Some symptomatic patients require surgical intervention, whereas prevention through measures targeting important causal pathways should avoid all morbidity and sequelae. Long-term statin use was associated with reduced GBD in a recent observational case–control design, but bias and confounding cannot be ruled out,^11^ and results of long-term randomized controlled trials are not yet available.

Although family-based genetic linkage studies of GBD have met with limited success, inbred mouse studies^12^ and human genome-wide association studies (GWASs)^13^ converged on one notable locus, the head-to-head gene pair *ABCG5/ABCG8*, which together encode a heterodimeric transporter responsible for apical cholesterol secretion from both hepatocytes and enterocytes. However, a systems biology view must be taken since liver cell homeostasis compensates lesser absorption (more secretion) with more endogenous cholesterol synthesis.

We therefore conducted a large-scale genetic association study of GBD. This was conducted in women sampled from the general population. This design avoided potential selection bias of genotypes; with high single-nucleotide polymorphism (SNP) density coverage including many loci already well established to be involved in lipid metabolism or obesity. We analyzed our data in relation to this knowledge and interpret the findings in relation to metabolic pathways relevant to GBD.

## Materials and methods

### Study design

Genetic association analyses were conducted in order to identify candidate SNPs associated with GBD. Fine mapping of causal loci and effects of lipids were also considered. Only women were tested as two of the cohorts analyzed included only women. Findings derived from this work will therefore be applicable only to women.

Study descriptions and phenotype definitions are shown in Supplementary Information.

Data Access for the NHLBI Candidate gene Association Resource (CARe). The NHLBI initiated the CARe to create a shared genotype/phenotype resource for analyses of the association of genotypes with phenotypes relevant to the mission of the NHLBI. The resource comprises nine cohort studies funded by the NHLBI including: Atherosclerosis Risk in Communities (ARIC), Cardiovascular Health Study (CHS), Cleveland Family Study (CFS), Coronary Artery Risk Development in Young Adults (CARDIA), Framingham Heart Study (FHS), Jackson Heart Study (JHS), Multi-Ethnic Study of Atherosclerosis (MESA) and the Sleep Heart Health Study (SHHS). A database of genotype and phenotype data was created that includes records for ~41 000 study participants with ~50 000 SNPs from >2000 selected candidate genes. Data from individual cohorts such as ARIC are available to the approved investigators upon submission of data requests through the dbGaP portal.

### Genotyping and quality control

Genotype quality control characteristics from the three data sets are described in Supplementary Table 2. Genomic control inflation factors (*λ*) were 1.00 for all three cohorts. Genotyping was performed using the Human cardiovascular disease (HumanCVD) BeadChip (Illumina, San Diego, CA, USA), also known as the ITMAT-Broad-CARe (IBC) array as previously described.^14^ The ARIC and BWHHS used the IBCv2 array, which contained up to 49 240 SNPs, whereas the WHI used the IBCv3 array, which contained up to 53 400 SNPs (containing the entire v2 content plus an additional ~4200 SNPs from updated metabolic GWAS findings in the literature from 2007 to 2008). All SNPs were clustered into genotypes using the Illumina Beadstudio software and subjected to quality control filters at the sample and SNP level, separately within each cohort. Samples were excluded for individual call rates <90%, gender mismatch and duplicate discordance. SNPs were removed for call rates <95% or Hardy–Weinberg disequilibrium *P*<10^–7^. Because of the low frequency SNPs included in the design, and the aim to capture low frequency variants of large effect across the large dataset, we filtered only on minor allele frequency (MAF) <0.01. Following identification of the *TTC39B* SNP rs581080 in WHI that was absent in IBCv2, all BWHHS samples were genotyped for rs581080 using the KASPar system (KBiosciences, Essex, UK).^15^

### Statistical analyses

Only women of European ancestry were included. For each of the three studies, we verified self-reported ethnicity by multidimensional scaling analysis of identity-by-state distances as implemented in PLINK,^16^ including HapMap panels as reference standards. After pruning of SNPs in linkage disequilibrium (LD; *r*^2^>0.3), Eigenstrat was used to compute principal components (PCs) on the subset of non-excluded individuals for use as covariates in the regression analyses to control for the influence of population admixture.^17, 18^

GBD association analysis was performed in each study using an additive genetic model. We performed association analyses using GBD case/control adjusted for age and additionally adjusted for PCs if population structure was evident. Analyses adjusting for BMI were also performed. The *λ* was calculated in each case–control study and used for within-study correction before meta-analysis. Meta-analysis was performed using a fixed-effect standard error-based approach using METAL (1). The Candidate gene Association Resource (CARe) IBC array studies,^19^ determined that after accounting for LD, the effective number of independent tests was ~20 500 for Europeans producing an experimental or ‘array-wide' statistical threshold of *P*=2.4 × 10^−6^, respectively, to maintain a false-positive rate of 5%,^20^ which has been employed in numerous studies to date.^21, 22, 23, 24^ We also highlight loci significantly associated at a more conventional genome-wide significant threshold of *P*<5.0 × 10^−8^.

Loci harboring marginally significant evidence for association at *P*<10^−5^ were examined for independent signals via regression analyses in PLINK.^16^ A term was added to the regression model including the lead SNP as a covariate, and SNPs within the same candidate gene, or ±200 kb if the candidate gene region was <200 kb, were evaluated for maintaining array-wide significance. HDL-adjusted meta-analysis and conditional analysis were performed in the WHI and ARIC cohorts in which individual-level genotype data were available. A locus-specific Bonferroni correction was applied to determine the significance of independent signals.

Regional association plots for the genomic regions associated with GBD were constructed using the online tool LocusZoom (http://csg.sph.umich.edu/locuszoom/). When drawing the plots, the genome build HG18 was selected to provide the genome coordinates of SNPs. 1000 Genome CEU was used as the LD population.

The variance explained (adjusted *R*^2^) by associated SNPs (defined to be with *P-*value<1 × 10^−4^ in the primary genetic association tests) was calculated within cohorts with individual-level genotype and phenotype data available (ARIC and WHI) using a linear regression model incorporating the adjustment by covariates of age and three PC's. The average variance explained, weighted by the sample size of each contributing study, is reported.

### Fine mapping of candidate loci

Fine mapping of candidate loci has been performed using an approach we previously developed (Sequential Sentinel SNP Regional Association Plot, SSS-RAP)^25^ (software available at http://apps.biocompute.org.uk/sssrap/sssrap.cgi). SSS-RAP, together with conditional analysis, was used to identify independent signals. In short, this method is biologically driven and considers the relationship between LD and linear/logistic regression for pairwise SNPs. We adopted the additive model, typical for genes with small effect, and transformed the effect of a sentinel SNP to the predicted effect for a possibly dependent SNP.

There are 77 SNPs in the *ABCG5/8* locus designed on the IBC array, thus we used locus-wide significance level of 0.05/77=6.49 × 10^−4^ as the cutoff for conditional analysis. In the first round conditional analysis, we took genotypes of the lead SNP (rs4953023:G>A) in the un-conditional meta-analysis, redid association tests by adjusting its genotypes as a covariate in each individual-level cohort, and meta-analyzed the results. Then in the second round, we extracted the lead SNP of the first round conditional analysis (rs4299376:G>T), took the combination of genotypes for SNPs rs4953023:G>A and rs4299376:G>T as a covariate, and repeated the above procedure. We performed third and fourth rounds similarly. Within the *ABCG5-8* region, 360 SNPs were analysed from the 1000 genomes database based on 26 different populations from many different worldwide locations (http://www.1000genomes.org/). A total of 118 SNPs remained after excluding SNPs in perfect LD (*r*^2^=1). A random subset of SNPs not in the meta-analysis was excluded, with the remaining SNPs analysed using SSS-RAP. The rationale of random exclusion was to keep a representative sample of the study. Using this approach, 10/118 SNPs remained significant as signals in the meta-analysis.

### Associations of lipid SNPs with GBD

Median −log_10_ *P*-values were analyzed for 65 loci previously reported to associate with plasma lipid levels^26^ in comparison with the distribution of median −log_10_*P*-values generated by 10 000 simulations using random sampling of 65 loci for each set of simulations. These Monte Carlo simulations were performed in R.

Representations of the histogram of median estimates were performed using the ‘hist' function in the basic package of R (http://svn.r-project.org/R/trunk/src/library/graphics/R/hist.R), and the quantile–quantile (QQ) plots were also performed in R, but using the ‘ggplot2' package (http://cran.r-project.org/web/packages/ggplot2/index.html).

## Results

### Meta-analysis

Seventeen SNPs with call rates >95% and Hardy–Weinberg disequilibrium (*P*>10^–7^) with MAF >0.01 showed significant association with GBD at a level of *P*≤2.4x10^−6^ in our meta-analysis (Table 1). Fourteen of these variants localized to the *ABCG5*/*ABCG8* locus (Table 1), including the strongest signal found in our study (with rs4953023:G>A having the strongest value, *P*=7.41x10^−47^). The three remaining SNPs corresponded to the genes *GCKR* and *TTC39B* (Table 1). A less stringent threshold of *P*≤10^−5^ revealed 18 more SNPs that showed marginally significant association with GBD at a level of *P*≤10^−5^ in our meta-analysis (Supplementary Table 3). One of these variants localized to the *ABCG5*/*ABCG8* locus. The 17 remaining SNPs corresponded to the genes *TTC39B*, *GCKR, C2orf16*, *CCL20*, *RUNX1*, *EIF2B4*, *MMP24*, *CYP4F2*, *RIPK1*, *ZNF512* and *GAA* (Supplementary Table 3). The effect sizes and standard errors are also shown in Table 1 and Supplementary Table 3.

As there are two versions of the IBC array used in this study, this resulted in missingness for some SNPs across the cohorts. Table 1 shows the concordance of the direction of the effect found in all three cohorts (ARIC, BWHHS and WHI). There is concordance of direction for nine *ABCG5*/*ABCG8* SNPs, discordance for none, and incomplete information for six (five of the six SNPS were present on the version 3 array only). With regard to the other genes, we found the same direction of effect in all three cohorts for SNPs in *GCKR, C2orf16*, *CCL20*, *RUNX1*, *MMP24, CYP4F2, RIPK1* and *ZNF512*. *TTC39B* showed incomplete information (Supplementary Table 3).

Results for HDL-adjusted meta-analysis are shown in Supplementary Table 4. In summary, after adjusting for HDL, all of the *ABCG5/8* SNPs with the exception of rs4148189:C>T and rs6544713:T>C are significant considering the array-wide significance level, whereas *TTC39B* SNPs are not significant considering the array-wide significance level. Most strong hit for this gene is rs686030:C>A, with *P*=0.0038. *GCKR* SNP rs1260326 it is still marginally significant (*P*=1.01 × 10^−5^ after the HDL adjustment).

SNPs with *P*-value <1 × 10^−4^ (37 SNPs in total) explain 0.0808 of the variance in ARIC (note 11 SNPs are not genotyped in ARIC), while they explain 0.0917 of the variance in WHI (one *ABCG5* SNP rs4148191:G>T was not genotyped in WHI).

### Conditional analyses and regional association plots

Our conventional conditional analysis detected four independent SNP effects in *ABCG8/5* rs4953023:G>A, rs4299376:G>T, rs6544718:T>C and rs6720173:G>C (Supplementary Table 6) A different method to perform conditional analyses (SSS-RAP), after setting rs4953023:G>A as a top hit (lowest *P*-value), identified three SNPs (rs10208987:T>G, rs4299376:G>T and rs3806470: G>A) as possible independent signals associated with GBD. The possible independence among these SNPs was supported by the low pairwise *r*^2^ values observed (*r*^2^<0.22).

High degrees of heterogeneity when combining the three cohorts and unavailability of individual-level data in BWHHS are possible causes to explain differences between conditional analysis and SSS-RAP results.

Regional association plots are shown in Supplementary Figures 4–15.

### LD involving loci significantly associated with GBD

LD results for loci significantly associated with GBD are shown in Supplementary Information.

### Analyses of loci in lipid pathway

**Figure 1:**
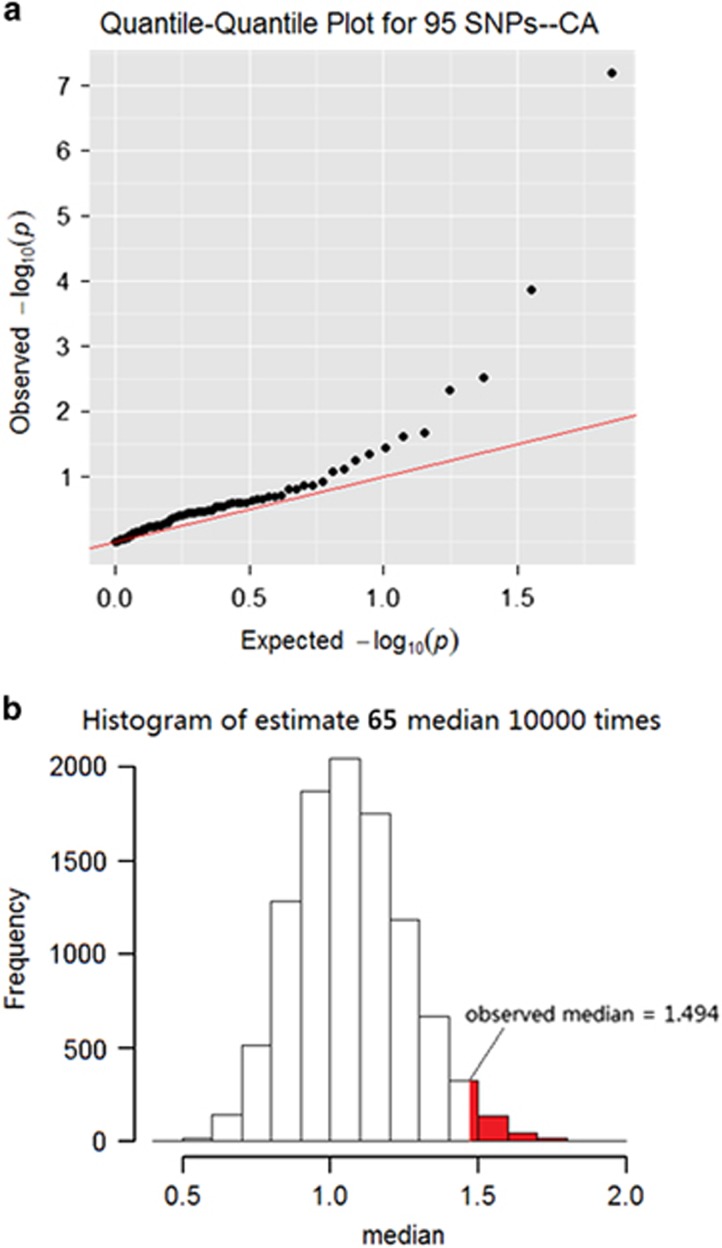
Pathway analysis relating GBD with SNPs associated with plasma lipid levels. (**a**) GBD QQ plot for SNPs representing 65 independent loci reported in a published study to influence plasma lipid levels. (**b**) Monte Carlo analysis of the median log10 *P*-value when sampling 65 SNPs at random from our IBC array meta-analysis (excluding SNPs with MAF <0.05), 10 000 random samplings. The observed median was 1.49. The median observed for the lipid set (**b**) was 1.06 (mean=1.07, SD=0.19).

From the 95 independent loci previously reported to be associated with blood lipid levels,^26^ it was possible to subject 65 to a test using IBC array data (Supplementary Table 5). Figure 1a shows the QQ plots relating the distribution of significant associations for GBD observed for SNPs representing 65 independent loci previously reported to associate with plasma lipid levels, compared with a random set. Out of 65 SNPs, 59 showed an observed *P*-value above the expected *P-*value in a QQ plot. The observed distribution (for lipid-associated loci) was significantly different than the expected distribution (for a random set). This is in accordance with a combined effect of these SNPs both on GBD and on plasma lipid levels variation.

Figure 1b shows results for Monte Carlo simulations analyzing the median −log_10_*P*-value of 10 000 samples of groups of 65 random SNPs in IBC. The observed median value for the 65 lipid-associated loci was 1.06 (mean=1.07, SD=0.19). A median −log_10_*P*-value greater than that observed for the lipid loci set was only observed in 1.7% of random samplings.

In our analysis, a handful of loci influencing either LDLc or triglycerides showed the most prominent associations with GBD. Specifically, these were rs4299376:G>T in *ABCG5/ABCG8* (see above); rs2081687:T>C in *CYP7A1* (our proxy rs8192870:T>G has an *r*^2^=0.806 with rs2081687:T>C, *P*=0.021 for GBD); rs5756931:A>G in *PLA2G6* (our proxy rs4820314:C>T *r*^2^=0.661, *P*=0.0022 for GBD); rs28385705:T>C in *PCSK9* (*P*=0.011) and rs483140:G>C in *LIPC* (*P*=0.0037). Details about all the associations are presented in Supplementary Table 3.

### Pathway analysis

Tissue expression and pathway rationale were evaluated to support the associated gene loci reported in Table 1. We consulted a recent survey of tissue expression by RNA sequencing^27^ for transcripts showing particularly relevant expression to GBD. We noted that *CCL20* (highest expression in gallbladder), *ABCG5*, *ABCG8*, *CYP4F2*, *GCKR*, *RUNX1* and *TTC39B* all showed predominant expression in hepatobiliary and gastrointestinal tissues, providing biological support for a role in GBD. Extending our analysis further, we used GeneGO Metacore (Thomson Reuters, New York, NY, USA) to evaluate the 13 genes for direct interaction with 142 genes previously linked to GBD in the literature by Medical Subject Headings terms. Five genes (*ABCG5*, *ABCG8*, *CCL20*, *RUNX1* and *CYP4F2*) showed direct interaction with genes linked to GBD by MESH in a network of directly interacting genes. Notably, the *RUNX1* gene interacts directly with 13 other GBD-linked genes, whereas *CCL20* interacts with three GBD-linked genes, suggesting multiple levels of biological support for both genes. Supplementary Figures 2 and 3 show a sub-network from this analysis based only on direct interactors of the genes reported in this study.

### Analysis of potential pharmacologic targets

Five of the novel genes reported here have available small molecule modulators (mainly inhibitory or binding) based on a query of the ChEMBL database (www.ebi.ac.uk/chembldb/). The genes are listed with the number of tool compounds in brackets: *CYP4F2* (7), *GAA* (drugged, 22256), *GCKR* (68), *RUNX1* (7437), *TPH* (4). As the differing number of tool compounds indicates, some of these targets are likely to be the focus of pre-existing drug development and projects may still be ongoing or terminated. Once molecular properties of these compounds are considered to have a favorable profile, particularly concerning the required direction of therapeutic effect, they could be investigated in animal models of GBD. Some specific candidate molecules are discussed further below.

### Other relevant phenotypes

Supplementary Table 4 shows the values observed for cases and controls in BWHHS, ARIC and WHI in relation to relevant covariates for the interpretation of our meta-analysis. Significant differences were observed between cases and controls for both age and BMI in all three cohorts, with the exception of WHI in relation to age. This is in accordance with the known relation between these variables and GBD. However, no significant differences were observed between cases and controls in relation to height, hormone replacement therapy and statin use. Significant differences were observed for diabetes status in ARIC and WHI but not in BWHHS.

## Discussion

GBD is a common disease, with >700 000 cholecystectomies performed in the United States alone per year at a cost of approximately $6.5 billion.^6^ This study, the largest genetic epidemiological analysis of GBD conducted to date, shows that a substantial number of genotypes, which associate with plasma lipid levels, also associate with risk of GBD in women of European ancestry. Taken together with some of their known functions, these data provide evidence of causal contributions of metabolic pathways involving both cholesterol, intermediary metabolism of fats, carbohydrates, and phospholipids in relation to GBD. Overall, this systems genetics analysis, by establishing causality of associations, shows that appropriate approaches to reduction of total cholesterol including reduction of carbohydrate and triglyceride substrate availability to the hepatocyte, should result in a decrease in risk of GBD.

Discussion about the relevance of phenotypic definitions of GBD based on questionnaires is shown in Supplementary Information.

From our examination of up to ~53 000 SNPs included in the IBC array analysis, the most prominent SNPs, which reached array-wide significance, were at the *ABCG5/ABCG8* locus (Table 1). Signals in this locus, at such small *P*-values, are reassuring as it is a known positive control for GBD. The high density of SNP coverage of this locus in IBC compared with conventional GWAS arrays, combined with the large number of cases, allowed us to show conclusively (based on the absence of LD and retention in a combined model for variable selection) the presence of several mutually independent signals in known functional sites. Our leading SNP, rs4953023:G>A (*P*=7.41 × 10^−47^, effect size=0.734) is in perfect LD with the previously reported rs11887534:G>C (NM_022437.2(ABCG8):c.55G>C (p.Asp19His). This amino-acid variant is believed to be a functional SNP, which increases cholesterol secretion and hence the risk of super-saturation of bile. The rs4299376:G>T variant, also in *ABCG8*, tags an independent effect, possibly reflecting an earlier report of NM_022437.2(ABCG8):c.161A>G (p.Tyr54Cys) (*r*^2^~0.2 between rs4299376:G>T and rs4148211:A>G in Europeans) on GBD in Taiwanese. Two further independent SNPs were identified in *ABCG5*, rs10439467:C>T in intron 10 and rs6720173:G>C NM_022436.2(ABCG5)c.1810C>G (p.Gln604Glu). The ABCG5/ABCG8 transporter is important in regulating biliary cholesterol. Our identification of further new alleles at this locus influencing gallstone risk emphasises that an approach, which might target cholesterol secretion directly, could be useful in GBD prevention, though adverse effects on TC and LDLc levels would need to be avoided or countered. However, it does not prove that reduction of total cholesterol will be successful, as sequestration anywhere away from the biliary tree might have no consequence for GBD risk. Furthermore, the impact of *ABCG5/ABCG8* is much greater in GBD than it is on LDLc levels. *ABCG8* does emerge in lipid GWAS but with a small effect, apparently because the hepatocyte compensates increased secretion by increasing cholesterol synthesis.

A pathway analysis of the gene loci reported here highlighted extensive connectivity between *ABCG5*, *ABCG8*, *RUNX1*, *CCL20* and nuclear hormone receptors (*PPARG*, *PPARD*, *LXRA* and *RAR*) with important roles in lipid homeostasis. The influence of thiazolidinedione drugs on gallstones has not been extensively studied, although two preclinical studies of pioglitazone conflict, a study by Al-Azzawi *et al.*^28^ showed a drug induced increase in gallbladder volume leading to increased gallstone formation in mice, whereas another study showed that the drug reduced gallstone formation in rats, by the action on retinol-binding protein-4.^29^ Our findings highlight the potential therapeutic importance of the network of interactions between members of the *PPAR* family, *ABCG5/8*, *CCL20* and *RUNX1*. We evaluated all the associated loci for evidence of small molecule druggability (Supplementary Methods). The protein products of several loci reported here are potentially druggable, but *RUNX1* (Runt-related transcription factor 1) may be of immediate interest in a novel target for GBD. *RUNX1* has an osteogenic role in the pathology of osteoarthritis, which was downregulated by a thienoindazole molecule (TD-198946) recently described by Yano *et al.*^30^ *RUNX1* may also have a role in atherosclerotic plaque formation.^31^ It is intriguing to speculate that *RUNX1* may have a similar role in the gallstone matrix in GBD.

Our pathway analysis relies on prior knowledge of gene function; however, we also describe potentially important associations in several highly novel genes of unknown function. A strong genetic signal is reported in *TTC39B* (rs686030:C>A), at array-wide significance, the first report implicating this locus in GBD. A variant in tetratricopeptide repeat domain 39B (*TTC39B*) at 9p22, rs581080:C>G, has been associated with levels of HDL cholesterol and with genotype-expression association in the Global Lipids Consortium.^26^ Lower *TTC39B* transcript levels and higher HDL cholesterol are evident in humans^32^ and knockdown of *TTC39B* in murine models also showed a clear increase in HDL.^26^ The function of *TTC39B* is unknown; however, the gene shows predominant gastrointestinal expression and the gallbladder was the second most highly expressed tissue after the skin, providing further support for a role of this novel gene in GBD. Our results showing that the *TTC39B* association is attenuated after HDL adjustment are consistent with HDL either mediating the association with GBD or acting as a confounder. Further studies are required to elucidate this.

Therapeutic implications of our findings are shown in Supplementary Information.

Discussion about our combined analysis of 65 independent loci for blood lipid levels and GBD is presented in Supplementary Information.

**Figure 2:**
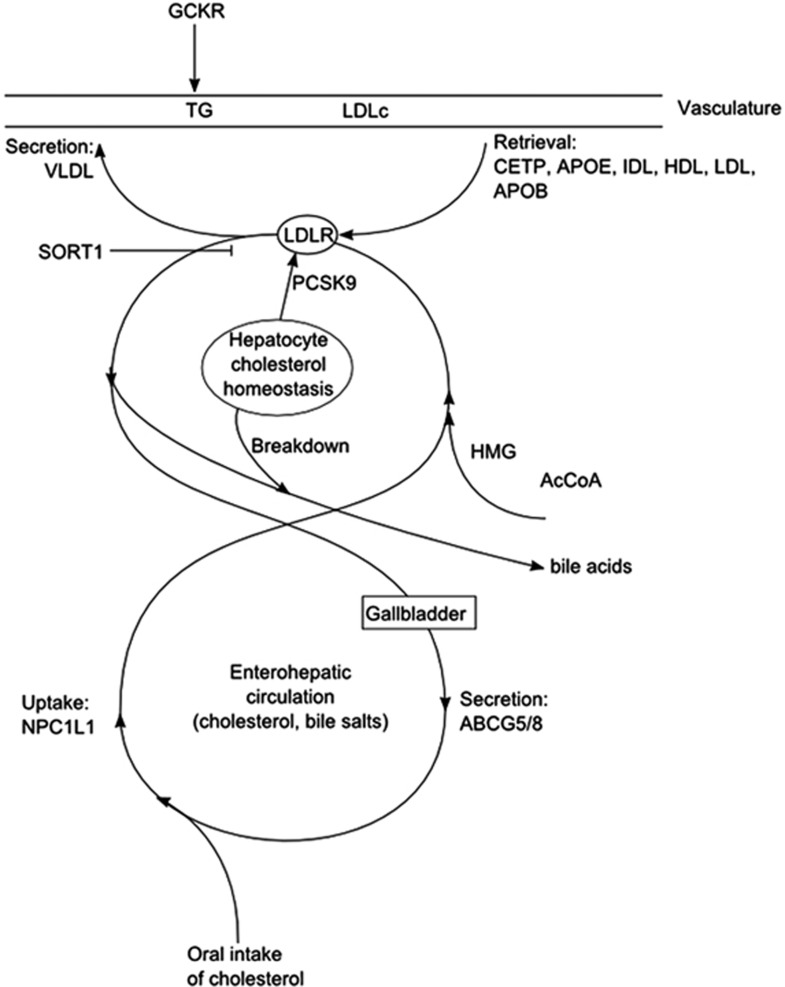
Gallbladder-centric view of cholesterol system. The cholesterol system can be described as three loops from a gallbladder-centric perspective. These are the enterohepatic cholesterol circulation, the loops of hepatocyte cellular homeostasis of cholesterol (and intermediary metabolism) and the total body status and turnover of lipids. Enhancing cholesterol breakdown, reducing synthesis and reducing reuptake from plasma should reduce gallstone risk.

An overall system, from the perspective of the gallbladder, can be described as three loops (Figure 2): first and most proximal, the enterohepatic cholesterol circulation; second, the loops of hepatocyte cellular homeostasis of cholesterol (and intermediary metabolism); and more distally, the total body status and turnover of lipids. Each known genetic perturbation represents an experiment of nature showing the likely directional outcome of an equivalent environmental or pharmacological intervention, similar to the causal role reported for *IL6R* in relation to coronary heart disease.^33^ It is evident from the above that enhancing cholesterol breakdown, reducing synthesis and reducing reuptake from plasma should reduce GBD risk, although the third strategy would not be attractive from a vascular perspective. It is notable that fibrate usage to reduce plasma triglycerides, increases GBD risk apparently through increasing cholesterol synthesis and hence flux through the biliary tree. The LIPC and GCKR data suggest that reducing substrate availability for endogenous cholesterol synthesis should also be effective in modifying GBD risk.

Overall, these systems genetic analyses show that regulation of hepatocyte cholesterol and intermediary metabolism exert causal effects in GDB risk in women. For pragmatic reasons, we only included women in our study and results may therefore only generalise to women. Further studies in cohorts of men with similar sample sizes would be required to extend our findings in men. Preventive measures, dietary and pharmacological, should meet with success considering the genetic evidence presented here that the observed associations are causal.

## Figures and Tables

**Table 1:** Association of SNPs with GBD in the ARIC, BWHHS and WHI IBC array studies

*Marker name*^a^	*A1*	*A2*	*Freq1*	*Effect*	*SE*	P*-value*	*Direction*	*HetChiSq*	*HetPVal*	*CHR*	*Hg18_bp*	*Gene*	*GeneAnno*
rs4953023	a	g	0.0657	0.7335	0.051	7.41 × 10^−47^	+++	7.219	0.02706	2	43927504	*ABCG8*	Intron
rs6756629	a	g	0.0651	0.6297	0.0682	2.54 × 10^−20^	+nn	0	1	2	43918594	*ABCG8*	Near-gene-5
rs4299376	t	g	0.6846	0.278	0.0318	2.40 × 10^−18^	+++	5.848	0.05371	2	43926080	*ABCG8*	Intron
rs10208987	t	g	0.9208	−0.3678	0.049	6.37 × 10^−14^	−−−	2.587	0.2743	2	43896639	*ABCG5*	Intron
rs10439467	t	c	0.0616	0.3825	0.0546	2.55 × 10^−12^	+++	8.025	0.01809	2	43901850	*ABCG5*	Intron
rs6720173	c	g	0.1585	0.2618	0.0377	3.81 × 10^−12^	+++	1.63	0.4426	2	43893905	*ABCG5*	Coding-nonsynon, cds-reference
rs6709904	a	g	0.8846	−0.3729	0.0546	8.10 × 10^−12^	−nn	0	1	2	43933828	*ABCG8*	Intron
rs4953019	a	g	0.0749	0.3266	0.0506	1.11 × 10^−10^	+++	2.523	0.2832	2	43896897	*ABCG5*	Intron
rs2278357	t	c	0.1607	0.2379	0.0374	2.10 × 10^−10^	+++	2.093	0.3512	2	43893343	*ABCG5*	Untranslated
rs10201851	t	c	0.8821	−0.2565	0.0427	1.82 × 10^−9^	−−−	3.601	0.1652	2	43900089	*ABCG5*	Intron
rs6544713	a	g	0.3125	−0.2385	0.0401	2.69 × 10^−9^	−nn	0	1	2	43927385	*ABCG8*	Intron
rs4148189	t	c	0.113	0.2446	0.0432	1.54 × 10^−8^	+++	3.423	0.1806	2	43901034	*ABCG5*	Intron
rs4148191	a	c	0.0721	0.5056	0.0948	9.52 × 10^−8^	n+n	0	1	2	43896408	*ABCG5*	Intron
rs4148196	c	g	0.8364	−0.2484	0.0475	1.73 × 10^−7^	−nn	0	1	2	43891418	*ABCG5*	3 downstream
rs1260326	t	c	0.4144	−0.1463	0.0293	5.88 × 10^−7^	−−n	3.152	0.2068	2	27584444	*GCKR*	Coding-nonsynon, cds-reference
rs686030	a	c	0.8581	0.271	0.0546	6.95 × 10^−7^	+nn	0	1	9	15294782	*TTC39B*	Intron
rs661048	a	g	0.8663	0.2664	0.0561	2.04 × 10^−6^	+nn	0	1	9	15287629	*TTC39B*	Intron

Abbreviations: ARIC, Atherosclerosis Risk in Communities; CHR, chromosome; GBD, gallbladder disease; IBC, ITMAT-Broad-CARe; SNP, single-nucleotide polymorphism.

Marker name is SNP name; A1 is allele 1 (ref allele); A2 is allele 2; effect is the beta coefficient; *P*-value. Direction: + indicates the same direction of effect; − indicates different direction; n indicates no data available for that particular SNP. Cohorts are in order, WHI/ARIC/BWHHS. HetChiSq=heterogeneity test statistic, HetPVal=heterogeneity *P*-value. Gene, gene name, GeneAnno, location of the SNP within the gene.

a Full names of these SNPs are rs4953023 (NT_022184.16:g.27700742G>A), rs6756629 (NT_022184.16:g.27691832G>A), rs4299376 (NT_022184.16:g.27699318G>T), rs10208987 (NT_022184.16:g.27669877T>G), rs10439467 (NT_022184.16:g.27675088C>T), rs6720173 (NT_022184.16:g.27667143G>C), rs6709904 (NT_022184.16:g.27707066A>G), rs4953019 (NT_022184.16:g.27670135G>A), rs2278357 (NT_022184.16:g.27666581C>T), rs10201851 (NT_022184.16:g.27673327T>C), rs6544713 (NT_022184.16:g.27700623T>C), rs4148189 (NT_022184.16:g.27674272C>T), rs4148191 (NT_022184.16:g.27669646C>A), rs4148196 (NT_022184.16:g.27664656C>G), rs1260326 (NT_022184.16:g.11361954T>C), rs686030 (NT_008413.19:g.15294784C>A), rs661048 (NT_008413.19:g.15287631G>A.
