# Supplementary material for: Lipids, obesity and gallbladder disease in women: insights from genetic studies using the cardiovascular gene-centric 50K SNP array
Source: Eur J Hum Genet. 2015 Apr 29;24(1):106–12. doi: 10.1038/ejhg.2015.63 (PMC4681116; doi:10.1038/ejhg.2015.63)

Supplementary methods

***Study design***

Genetic association analyses were conducted in order to identify candidate SNPs associated with GBD. Fine mapping of causal loci and effects of lipids were also considered. Only women were tested since two of the cohorts analysed included only women. Findings derived from this work will therefore be applicable only to women.

***Study descriptions***

The Atherosclerosis Risk in Communities Study (ARIC), is a prospective community based epidemiologic study designed to primarily investigate atherosclerosis and cardiovascular (CV) risk factors ^1^. 15,792 individuals were recruited in total between 1987-89, and re-examinations took place triennially through 1996-98.

The British Women's Heart and Health Study (BWHHS) is a prospective cohort study of over 4,000 British women between the ages of 60 and 79 years at baseline ^2^. Extensive measurements of CV risk factors and detailed information about lifestyle and medical history of heart disease are available.

Women's Health Initiative (WHI) is a National Insitutes of Health (NIH) study aiming to prevent diseases in women ^3^. The original WHI study included 161,808 postmenopausal women with ages between 50 and 79 years, enrolled between 1993 and 1998. One of the components of the WHI is an Observational Study, conducted at 40 Clinical Centres in the U.S. ^3^, which analyses the medical history and health habits of 93,676 women.

Additional study descriptors from each of the contributing studies are presented in Supplementary Table 1. Only women and individuals of European origin were included in the analyses (see Supplementary Table 1). All participating studies obtained informed consent for DNA analysis and appropriate approval from local institutional review boards.

***Phenotype Definitions***

GBD was defined as follows: (a) in the ARIC cohort participants were asked in 1993-96, "Have you ever been diagnosed by a doctor as having gallstones or a gallbladder attack? (Yes/No)" (see www.cscc.unc.edu/aric/pubuse/form/cohort/exam3/AMHA.pdf) (b) in the BWHHS, GBD was self reported on questionnaires from doctor diagnosis (Have you ever been told by a doctor that you had GBD: Yes/No); and (c) in the WHI study, prevalent GBD was self reported by the participant at baseline exam as a prior history of GBD. The participant was asked "Did a doctor ever say that you had gallbladder disease or gallstones?" Incident GBD was reported during the prospective WHI follow-up period as a first instance of GBD. The question for incidence was "Has a doctor told you that you have gallbladder disease or gallstones?"

Characteristics of the three cohorts of women are shown in Supplementary Table 1. The mean ages of participants at baseline were 53.9years (yrs) (SD=5.7yrs), 68.8yrs (SD=5.5yrs) and 68.0yrs (SD=6.6yrs), respectively, in ARIC, BWHHS and WHI. Numbers of cases and controls are also shown in Supplementary Table 1. 18.3%, 9.2% and 27.6% of the participants in ARIC, BWHHS and WHI, respectively had GBD. In total for this study we included 3,216 women with GBD and 12,025 controls.

***Genotyping and quality control***

Genotype quality-control characteristics from the three datasets are described in Supplementary Table 2. Genomic control inflation factors (λ) were 1.00 for all three cohorts. Genotyping was performed using the Human cardiovascular disease (HumanCVD) BeadChip (Illumina, San Diego, CA), also known as the ITMAT-Broad-CARe (IBC) array as previously described ^4^. The ARIC and BWHHS used the IBCv2 array which contained up to 49,240 SNPs while the WHI used the IBCv3 array which contained up to 53,400 SNPs (containing the entire v2 content plus an additional ~4,200 SNPs from updated metabolic GWAS findings in the literature from 2007 to 2008). All SNPs were clustered into genotypes using the Illumina Beadstudio software and subjected to quality control filters at the sample and SNP level, separately within each cohort. Samples were excluded for individual call rates<90%, gender mismatch and duplicate discordance. SNPs were removed for call rates <95% or Hardy-Weinberg disequilibrium p<10^–7^. Due to low frequency SNPs included in the design, and the aim to capture low frequency variants of large effect across the large dataset, we filtered only on minor allele frequency (MAF)<0.01. Following identification of the TTC39B SNP rs581080 in WHI that was absent in IBCv2, all BWHHS samples were genotyped for rs581080 using the KASPar system (KBiosciences, Essex, UK) ^5^ .

**Pathway Analysis**

Associated genes were reviewed for evidence linking them to GBD-related phenotypes using PubMed and “Gall Bladder Disease” as a Medical Subject Headings (MeSH) term. MESH is a controlled vocabulary created by the National Library of Medicine (NLM) to index journal articles and books in the life sciences. All articles in MEDLINE have been annotated with MeSH by NLM curators or designees, offering a sensitive measure of correlation between biological traits and genes in the literature ^6^. At a pathway level, we used GeneGo Metacore (Thomson Reuters) to construct a custom GBD network based on direct interactions between 142 genes annotated to the “Gall Bladder Disease” MESH term. The Metacore database is a large commercial database of curated human, rat and mouse gene and protein interactions individually evidenced in the literature ^7^. This allowed us to construct a core network of interacting genes that are each individually linked to gall bladder disease and related traits. We used this network as a tool to investigate direct interactions between the GBD gene network and the genes in the GBD loci reported here. By combining network data with data on gene expression and variant function we were able to prioritize genes based on their level of support with respect to GBD phenotypes.

**Analysis of pharmacologic targets**

We annotated associated genes with available public information concerning potential druggability – that is, the potential for modulation of the protein target by a water-soluble small molecule drug. Druggable proteins usually contain a defined binding pocket or active site, which could act as a site of action (pharmacophore) for an orally bioavailable small molecule drug. We grouped proteins into four druggability classes, based on complementary annotations of the potentially druggable genome and publicly available databases of small molecules ^8, 9^. Targets in class 1 are already drugged with a marketed drug recorded in DrugBank (www.drugbank.ca); class 2 have small molecules recorded in Chembl, (www.ebi.ac.uk/chembl) which may include compounds in current development within pharmaceutical companies, and could be used as tools in animal and cellular models; class 3 are homologous to class 1 or class 2 targets; class 4 are predicted to contain a potentially druggable pharmacophore based on de novo structure-based druggability prediction using the DogSiteScorer tool ^10^, which provides binding site prediction, analysis and druggability.

Supplementary Results

***LD involving loci significantly associated with GBD***

Supplementary Figure 1A shows LD (measured by *r^2^*) between each of the *ABCG8* SNPs significantly associated with GBD in our study and rs4953023:G>A. This SNP is in perfect LD with the coding variant rs11887534:G>C (representing an Aspartic acid to Histidine change at amino acid residue 19: D19H (c.55G>C (p.(Asp19His)), predicted possibly damaging by PolyPhen ^30^) that was previously associated with cholesterol gallbladder stones in previous GWAS and linkage studies ^31, 32^. The rs11887534:G>C variant failed the IBC array design stage and is thus not present in this dataset. Only rs4953023:G>A (in intron 3 of *ABCG8*), which yields both the highest significance (*P*=7.41x10^-47^) and the highest effect size (regression coefficient ß=0.734) in our study, shows perfect LD (r^2^=1) with rs11887534:G>C in Europeans in HapMap. Our conditional analyses and LD observations support rs4953023:G>A as a genetic risk factor for GBD and is likely independent of the other *ABCG8* SNPs that are significantly associated with GBD in our study. One of these *ABCG8* SNPs, rs4299376:G>T, was found to be significantly associated with GBD (*P*=2.40x10^-18^) in our study with a lower effect size (ß=0.278) observed. In relation to rs4148211:A>G (a Cysteine to Tyrosine change at residue 54: c.161A>G (p.(Tyr54Cys), predicted benign by PolyPhen) there is a reported association with this coding SNP for GBD in a Taiwanese study, with an r^2^ between rs4148211:A>G and rs4299376:G>T of 0.209 ^33^. However, in HapMap-CEU (U.S. residents with northern and western European ancestry), the observed LD between this pair of SNPs is r^2^=0.161, D’=1 (HapMap, http://hapmap.ncbi.nlm.nih.gov/index.html.en). Our results support that these two SNPs may be in one LD block and may represent the same functional mechanism or (unknown) causal SNP.

Supplementary Figure 1B shows LD between rs10439467:C>T in *ABCG5*, and other *ABCG5*/*8* SNPs. Rs10439467:C>T appears to mark a novel independent effect which does not appear to have been directly represented or captured in earlier chip-based analyses. There is essentially no LD between this SNP and rs6720173:G>C :c.1810C>G, (p.(Gln604Glu) (r^2^=0.095 in Europeans in HapMap), which is the only reported coding SNP for GBD in *ABCG5*. This confirmed its independence of effect on GBD.

Supplementary Figure 1C shows LD between each of the SNPs in *ABCG5* with rs6720173:G>C :c.1810C>G, (p.(Gln604Glu) (*P*=3.81x10^-12^, ß=0.262). One significant SNPs rs2278357:C>T (, *P*=2.10x10^-10^, ß=0.238) is LD (r^2^=1) with c.1810C>G, (p.(Gln604Glu) (predicted benign by PolyPhen). Five other genome-wide significant SNPs in the *ABCG5* region were no longer retained as independent predictors, respectively rs10208987:T>G (*P*=6.37x10^-14^, ß=-0.368, r^2^=0.141), rs10439467 (*P*=2.55x10^-12^, ß=0.383, no LD information), rs4953019:G>A (*P*=1.11x10^-10^, ß=0.327, r^2^=0.209), rs10201851:T>C (*P*=1.82x10^-9^, ß=-0.257, r^2^=0.160) and rs4148189:C>T (*P*=1.54x10^-8^, ß=0.245, r^2^=0.200).

There is strong LD (r^2^=0.706 D’=1.000) between the TTC39B SNP rs581080:C>G which was previously associated with HDL and TC levels ^29^ and our main finding from IBCv3 (rs686030:C>A). We note that rs581080:C>G did not pass QC in the production phase for the IBCv3 design, so was not included in the array.

**Adjustment for BMI**

Results obtained after adjusting for BMI were similar to those obtained after multivariate analysis adjusting for BMI (data not shown). The SNPs associated with GBD were the same in both instances for associations showing P<10^-6^, although the P values were one or two orders of magnitude higher when BMI was adjusted for.

Supplementary Discussion

Our phenotypic definitions are based on questionnaires for GBD and not on abdominal ultrasound. However, the principal association on *ABCG5/8* for GBD in our study corresponds with *ABCG5/8* association found previously from GWAS of gallstone disease, consistent with the close relationship between GBD and gallstones. On the other hand, it has been suggested that analyses of self-reported GBD and other co-morbid illnesses may limit the generalisation of results obtained in a population. In particular, it has been reported that this may lead to underreporting the incidence of GBD in populations such as Mexican origin population ^11^. However, there is no evidence suggesting that self-reported medical data has a major effect on genetic association results. A recent study of 50 medical phenotypes analysed on over 20,000 genotyped individuals replicated over 180 previously reported associations ^12^.

The existence of strong LD between the TTC39B SNP rs581080 ^13^ and our main finding (rs686030) associate these two variants and the effects of lipids levels and GBD. This finding is potentially relevant for therapeutic development. Statins and ezetimibe, two validated drug targets for hyperlipidaemia treatment involve HMGCR and NPC1L1 respectively ^13^. Likewise, the bridge between lipids and GBD represented by TTC39B and our earlier findings in the known lipid homeostasis pathways could be instrumental for the characterisation of the mechanism of action of GBD and the identification of novel therapeutic targets for this disease.

The IBC arrays are primarily gene centric around ~2,000 loci and have deeper coverage than conventional GWAS arrays in many CVD related loci. This is the case for the *ABCG5/ABCG8* locus, where there are 45 SNPs available out of ~45,000 (the locus was tagged to cover 2% minor allele frequencies in all three HapMap populations and all non-synonymous SNPs that could be designed), with genotypic information. This affords powerful secondary analyses, including conditional and fine mapping analyses to infer additional possible causal loci.

Secondly, we examined collections of loci recently reported from very large scale meta-GWAS of the traits of blood lipid levels, BMI, waist-hip-ratio and height ^13, 14^. Our null hypothesis was that in QQ plots and compared with appropriate random sampling of equivalent numbers of SNPs in the IBC array, the distribution of significance values would be no different for a trait-selected set of SNPs compared with a random set. However, considering the association of gallstones with obesity and their constitution of cholesterol, we hypothesised either a general difference of distribution, and/or that the leading SNPs for the trait would also show nominal significance for GBD. We did not expect height SNPs generally to affect GBD risk.

Our combined analysis of 65 independent loci for blood lipid levels and GBD, indicates that a large number of loci influencing blood lipid levels also influence GBD risk. We then divided by principal trait affected, LDLc, total cholesterol, HDLc or triglycerides. We noted that a handful of loci influencing either LDLc or triglycerides, showed the most prominent associations with GBD (rs4299376 in *ABCG5/ABCG8*; rs2081687 in *CYP7A1*; rs1260326 in *GCKR*; rs5756931 in *PLA2G6*; rs2479409 in *PCSK9* and rs1532085 in *LIPC*). *CYP7A1* encodes 7alpha hydroxylase, a rate limiting step in the breakdown of cholesterol to bile acids in the hepatocyte. Bile acids also assist in keeping cholesterol in emulsion in bile. PCSK9 participates in the regulation of LDL receptor internalization in the hepatocyte ^15^ and *GCKR* encodes glucokinase regulator. In the 1970s, the reciprocal steps (futile cycle) between glucose and glucose-6-phosphate (via glucokinase and glucose-6-phosphatase) were recognised to be a potentially sensitive and major mechanism of regulation of intermediary metabolism in the liver with the proposal that there must be either a regulator or compartmentalization ^16^. In the 1980s such a regulator was discovered and indeed GCKR exerts its influence by sequestering glucokinase to the nucleus when glucose is low ^17^. *GCKR* has also emerged in GWAS of type 2 diabetes (T2D) as well as plasma triglycerides. *GCKR* effectively represents a genocopy of a high/low carbohydrate diet, ultimately also influencing substrate availability for endogenous cholesterol synthesis. Cholesterol breakdown, cholesterol secretion, receptor-mediated cholesterol internalization and endogenous cholesterol synthesis represent four mechanisms for hepatocyte cholesterol homeostasis and it is notable that genetic influences on these factors are the most prominent in relation to GBD. In each instance, the plasma cholesterol or triglyceride raising allele is the risk allele for GBD. By contrast, some loci with very strong effects on blood lipids showed little significance for GBD, for example *LPL* (triglycerides), *CETP* (HDLc), with *SORT1* (LDLc) and *APOE* (lipoprotein particle turnover, LDLc, TG, HDLc). It is notable that these are more remote from hepatocyte cholesterol regulation, *SORT1* influences very LDL (VLDL) secretion and *LPL* and *CETP* acting in the vasculature. Ezetimibe has a direct effect through diminishing cholesterol levels in the liver. This drug acts directly on NPC1L1, a protein which acts together with ABCG5/G8 in the modulation of cholesterol levels ^18^. *NPC1L1* genetic variation has been associated with lipid levels ^13^, but we did not find any evidence of association with GBD in our study. However, LIPC (hepatic lipase) may have some effect on GBD risk. Lastly, PLA2G6 (also known as PNPLA9) draws attention to phospholipids. It encodes a calcium-independent phospholipase which is widely expressed. Phospholipids are in molar excess over cholesterol as are bile acids in normal bile and as amphipathic molecules help to ensure an emulsion of mixed micelles and vesicles combining aqueous and hydrophobic phases. It is possible that PLA2G6 acts directly on bile content, otherwise through membrane or fatty acid regulation and triglyceride turnover.

**Web Resources**

The URLs for data presented in the manuscript are as follows:

ChEMBL, http://www.ebi.ac.uk/chembl

DrugBank, http://www.drugbank.ca

Supplementary Table 1. Phenotypic characteristics of BWHHS in relation to age, BMI, height, hormone replacement therapy (HRT), statin use and diabetes. P values result from comparing observations for cases and controls.

|  | BWHHS | ARIC | WHI |
| --- | --- | --- | --- |
| **Cases/Controls**  **(total)** | 845 / 3,763 (4,608) | 284 / 2,794 (3,078) | 2,078 / 5,468 (7,555) |
| **Age: mean years (SD)** | Cases: 69.75 (5.40)  Controls: 68.67 (5.50)  Combined: 68.77 (5.50)  P = 0.0016 | Cases: 54.51 (5.72)  Controls: 53.72 (5.65)  Combined: 53.86 (5.67)  P = 0.0003 | Cases: 67.77 (6.53)  Controls: 68.02 (6.61)  Combined: 67.95 (6.59)  P = 0.1403 |
| **BMI: mean (SD)** | Cases: 29.30 (5.66) Controls: 27.32 (4.76)  Combined: 27.51 (4.88)  P < 0.0001 | Cases: 28.67 (6.26)  Controls: 26.04 (5.09)  Combined: 26.52 (5.42)  P < 0.0001 | Cases: 27.58 (5.88)  Controls: 30.34 (6.7)  Combined: 28.34 (6.24)  P < 0.0001 |
| **Height: mean (SD)** | Cases: 158.2 (6.29) Controls: 159.0 (6.08) Combined: 158.9 (6.10)  P = 0.0612 | Cases: 162.03 (5.88) Controls: 162.17 (5.91) Combined: 162.14 (5.90)  P = 0.9913 | Cases: 161.00 (6.40) Controls: 161.11 (6.35) Combined: 161.08 (6.36)  P = 0.9920 |
| **HRT: proportion ever taken** | Cases: 0.211 Controls: 0.228 Combined: 0.227  P = 0.4289 | - | - |
| **Statin use: proportion ever taken** | Cases: 0.074 Controls: 0.069 Combined: 0.069  P = 0.7634 | Cases: 0.040 Controls: 0.031 Combined: 0.033  P = 0.1636 | Cases: 0.110 Controls: 0.103 Combined: 0.105  P = 0.3586 |
| **Diabetes: proportion** | Cases: 0.127 Controls: 0.093 Combined: 0.096  P = 0.0698 | Cases: 0.183 Controls: 0.087 Combined: 0.105  P = <.0002 | Cases: 0.116 Controls: 0.060 Combined: 0.076  P = <.0002 |

P values were computed by a t test, used to compare the means of two groups for age, BMI and height. P values were computed by testing the difference between two independent proportions for HRT, statin use and diabetes.

Supplementary Table 2: Summary of the quality-control filters applied to samples genotyped in this GBD study

|  | ARIC | BWHHS | WHI |
| --- | --- | --- | --- |
| Initial number of SNPs | 49094 | 46176 | 53831 |
| SNPs with geno <95% | 264 | 4 | 1570 |
| HWE (P < 1 x 10^-7^) | 251 | 75 | 459 |
| Final number of SNPs | 45578 | 46097 | 51802 |
| Genotyping success rate | 99.87% | 99.82% | 99.00% |

Supplementary table 3. Marginal associations at P<10^-5^ and P>2.4x10^-6^

| MarkerName | A1 | A2 | Freq1 | Effect | StdErr | P-value | Direction | HetChiSq | HetPVal | CHR | Hg18_bp | Gene | GeneAnno |
| --- | --- | --- | --- | --- | --- | --- | --- | --- | --- | --- | --- | --- | --- |
| rs585002 | a | g | 0.1947 | -0.222 | 0.0476 | 3.13E-06 | -nn | 0 | 1 | 9 | 15283513 | TTC39B | intron |
| rs780094 | t | c | 0.4042 | -0.1366 | 0.0294 | 3.40E-06 | --- | 3.005 | 0.2225 | 2 | 27594741 | GCKR | intron |
| rs780093 | t | c | 0.3987 | -0.1363 | 0.0297 | 4.35E-06 | --- | 2.535 | 0.2815 | 2 | 27596107 | GCKR | intron |
| rs1919128 | a | g | 0.7268 | 0.15 | 0.0328 | 4.69E-06 | +++ | 0.255 | 0.8804 | 2 | 27655263 | C2orf16 | coding |
| rs4077440 | a | g | 0.4291 | -0.1661 | 0.0371 | 7.65E-06 | -nn | 0 | 1 | 2 | 43934546 | ABCG8 | intron |
| rs13389224 | t | c | 0.4367 | -0.1231 | 0.029 | 2.21E-05 | --- | 1.842 | 0.398 | 2 | 228377279 | CCL20 | unknown |
| rs8134380 | a | t | 0.5536 | 0.1219 | 0.029 | 2.62E-05 | +++ | 1.316 | 0.5179 | 21 | 35144009 | RUNX1 | intron |
| rs638491 | a | g | 0.1667 | -0.2097 | 0.0502 | 2.96E-05 | -nn | 0 | 1 | 9 | 15280012 | TTC39B | intron |
| rs7586601 | a | g | 0.5689 | -0.1202 | 0.029 | 3.34E-05 | --+ | 4.53 | 0.1038 | 2 | 27438170 | EIF2B4 | 5upstream |
| rs2425019 | a | g | 0.5442 | -0.1176 | 0.0288 | 4.41E-05 | --- | 6.473 | 0.03931 | 20 | 33282831 | MMP24 | intron |
| rs3093207 | t | c | 0.6526 | 0.1229 | 0.0304 | 5.40E-05 | +++ | 3.979 | 0.1368 | 19 | 15849658 | CYP4F2 | 3downstream |
| rs3093216 | a | g | 0.6527 | 0.1225 | 0.0304 | 5.49E-05 | +++ | 4.263 | 0.1187 | 19 | 15848737 | CYP4F2 | 3downstream |
| rs471364 | a | g | 0.8852 | 0.238 | 0.0597 | 6.63E-05 | +nn | 0 | 1 | 9 | 15279578 | TTC39B | intron |
| rs4959774 | a | g | 0.8932 | 0.1902 | 0.0479 | 7.08E-05 | +++ | 0.315 | 0.8544 | 6 | 3021335 | RIPK1 | 5upstream |
| rs2068834 | t | c | 0.7124 | 0.1269 | 0.0321 | 7.86E-05 | +++ | 0.426 | 0.8083 | 2 | 27693043 | ZNF512 | intron |
| rs12450199 | a | c | 0.6171 | 0.1163 | 0.0296 | 8.46E-05 | ++- | 3.749 | 0.1534 | 17 | 75691187 | GAA | untranslated |
| rs13472 | a | g | 0.3793 | 0.1151 | 0.0293 | 8.75E-05 | ++- | 3.65 | 0.1612 | 2 | 27453743 | EIF2B4 | locus,untranslated |
| rs2293572 | c | g | 0.6201 | -0.1142 | 0.0293 | 9.99E-05 | --+ | 3.63 | 0.1629 | 2 | 27582281 | GCKR | intron |

Marker Name = SNP name; A1 = Allele 1 (ref allele), A2 = Allele 2, Effect = beta coefficient; StdErr = standard error; P value; Direction: + indicates the same direction of effect, - indicates different direction, n indicates no data available for this SNP. Cohorts are in order, WHI/ARIC/BWHHS. HetChiSq = Heterogeneity test statistic, HetDf = Heterogeneity test statistic degrees of freedom, HetPVal = Heterogeneity p-value, CHR = chromosome; BP = location in base pairs, Gene = gene name, GeneAnno = location of the SNP within the gene

**Supplementary Table 4 HDL-adjusted meta-analysis testing association of SNPs with GBD in the WHI and ARIC** IBC array studies

| MarkerName | A1 | A2 | Freq1 | Effect | StdErr | P-value | Direction | HetChiSq | HetPVal | CHR | Hg18_bp | Gene | GeneAnno |
| --- | --- | --- | --- | --- | --- | --- | --- | --- | --- | --- | --- | --- | --- |
| rs4953023 | a | g | 0.0670 | 0.7220 | 0.0665 | 1.83x10^-27^ | ++ | 2.012 | 0.1561 | 2 | 43927504 | *ABCG8* | intron |
| rs6756629 | a | g | 0.0651 | 0.6339 | 0.0946 | 2.05x10^-11^ | +n | 0.000 | 1 | 2 | 43918594 | *ABCG8* | near-gene-5 |
| rs6720173 | c | g | 0.1608 | 0.2842 | 0.0488 | 5.57x10^-9^ | ++ | 0.214 | 0.6434 | 2 | 43893905 | *ABCG5* | "coding-nonsynon,cds-reference" |
| rs4299376 | t | g | 0.6862 | 0.2314 | 0.0408 | 1.4x10^-8^ | ++ | 2.281 | 0.1309 | 2 | 43926080 | *ABCG8* | intron |
| rs10208987 | t | g | 0.9196 | -0.3590 | 0.0647 | 2.88x10^-8^ | -- | 2.129 | 0.1446 | 2 | 43896639 | *ABCG5* | intron |
| rs2278357 | t | c | 0.1622 | 0.2677 | 0.0485 | 3.41x10^-8^ | ++ | 0.287 | 0.5921 | 2 | 43893343 | *ABCG5* | untranslated |
| rs10439467 | t | c | 0.0620 | 0.4011 | 0.0727 | 3.42x10^-8^ | ++ | 6.991 | 0.0082 | 2 | 43901850 | *ABCG5* | intron |
| rs4148191 | a | c | 0.0721 | 0.5074 | 0.0961 | 1.31x10^-7^ | n+ | 0.000 | 1 | 2 | 43896408 | *ABCG5* | intron |
| rs6709904 | a | g | 0.8846 | -0.3832 | 0.0752 | 3.46x10^-7^ | -n | 0.000 | 1 | 2 | 43933828 | *ABCG8* | intron |
| rs4953019 | a | g | 0.0760 | 0.3311 | 0.0671 | 8.01x10^-7^ | ++ | 1.978 | 0.1596 | 2 | 43896897 | *ABCG5* | intron |
| rs10201851 | t | c | 0.8812 | -0.2697 | 0.0559 | 1.38x10^-6^ | -- | 3.262 | 0.07091 | 2 | 43900089 | *ABCG5* | intron |

Marker Name = SNP name; A1 = Allele 1 (ref allele), A2 = Allele 2, Effect = beta coefficient; StdErr = standard error; P value; Direction: + indicates the same direction of effect, - indicates different direction, n indicates no data available for this SNP. Cohorts are in order, WHI/ARIC. HetChiSq = Heterogeneity test statistic, HetDf = Heterogeneity test statistic degrees of freedom, HetPVal = Heterogeneity p-value, CHR = chromosome; BP = location in base pairs, Gene = gene name, GeneAnno = location of the SNP within the gene

**Supplementay Table 5. Associations between GBD and 65 independent loci previously reported to be associated with lipid levels (Teslovich et al. Nature 2010;466:707-713).**

| rsID | Locus | lipids effect size | CA effect size | direction | leading trait | CA p value | MAF | proxy | r^2^ with proxy |
| --- | --- | --- | --- | --- | --- | --- | --- | --- | --- |
| rs1084651 | *LPA* | 1.95 | 0.0443 | same direction | HDL | 0.2921 | 0.16 | (A) | (A) |
| rs11869286 | *STARD3* | -0.48 | -0.0176 | same direction | HDL | 0.5955 | 0.34 | (A) | (A) |
| rs12967135 | *MC4R* | -0.42 | 0.0333 | opposite direction | HDL | 0.5422 | 0.23 | rs1943226 | 0.188 |
| rs1532085 | *LIPC* | 1.45 | -0.1122 | opposite direction | HDL | 0.03656 | 0.39 | (A) | (A) |
| rs16942887 | *LCAT* | 1.27 | -0.0259 | opposite direction | HDL | 0.7018 | 0.12 | rs2292318 | 0.915 |
| rs1800961 | *HNF4A* | -1.88 | 0.1497 | opposite direction | HDL | 0.0848 | 0.03 | (A) | (A) |
| rs1883025 | *ABCA1* | -0.94 | 0.0031 | opposite direction | HDL | 0.9312 | 0.25 | (A) | (A) |
| rs2652834 | *LACTB* | -0.39 | 0.0507 | opposite direction | HDL | 0.1896 | 0.2 | rs11071721 | 0.442 |
| rs2972146 | *IRS1* | 0.46 | 0.0329 | same direction | HDL | 0.3256 | 0.37 | rs2943634 | 0.774 |
| rs3136441 | *LRP4* | 0.78 | -0.0428 | opposite direction | HDL | 0.3594 | 0.15 | rs2070850 | 1 |
| rs3764261 | *CETP* | 3.39 | 0.0259 | same direction | HDL | 0.444 | 0.32 | (A) | (A) |
| rs386000 | *LILRA3* | 0.83 | -0.0491 | opposite direction | HDL | 0.2069 | 0.2 | rs103294 | 0.832 |
| rs4129767 | *PGS1* | -0.39 | -0.0364 | same direction | HDL | 0.2487 | 0.49 | rs4082919 | 0.967 |
| rs4846914 | *GALNT2* | -0.61 | -0.0608 | same direction | HDL | 0.05724 | 0.4 | (A) | (A) |
| rs6065906 | *PLTP* | -0.93 | 0.071 | opposite direction | HDL | 0.07487 | 0.18 | rs6073952 | 0.877 |
| rs7134375 | *PDE3A* | 0.4 | 0.0187 | same direction | HDL | 0.5553 | 0.42 | rs10841495 | 0.243 |
| rs7134594 | *MVK* | -0.44 | 0.0057 | opposite direction | HDL | 0.9127 | 0.47 | (A) | (A) |
| rs7241918 | *LIPG* | -1.31 | -0.0521 | same direction | HDL | 0.2255 | 0.17 | rs2156552 | 0.948 |
| rs7255436 | *ANGPTL4* | -0.45 | -0.036 | same direction | HDL | 0.253 | 0.47 | rs2278236 | 1 |
| rs838880 | *SCARB1* | 0.61 | -0.0323 | opposite direction | HDL | 0.352 | 0.31 | rs838878 | 0.961 |
| rs11220462 | *ST3GAL4* | 1.95 | -0.0629 | opposite direction | LDL | 0.2014 | 0.14 | rs8177375 | 0.121 |
| rs1367117 | *APOB* | 4.05 | -0.0311 | opposite direction | LDL | 0.358 | 0.3 | (A) | (A) |
| rs1800562 | *HFE* | -2.22 | 0.016 | opposite direction | LDL | 0.801 | 0.06 | (A) | (A) |
| rs2479409 | *PCSK9* | 2.01 | 0.0776 | same direction | LDL | 0.02155 | 0.3 | (A) | (A) |
| rs3757354 | *MYLIP* | -1.43 | -0.0129 | same direction | LDL | 0.8653 | 0.22 | rs7770341 | 0.157 |
| rs4299376 | *ABCG5/8* | 2.75 | 0.194 | same direction | LDL | 6.44E-08 | 0.3 | (A) | (A) |
| rs4420638 | *APOE* | 7.14 | 0.0002 | same direction | LDL | 0.9977 | 0.17 | (A) | (A) |
| rs6029526 | *TOP1* | 1.39 | -0.0186 | opposite direction | LDL | 0.5594 | 0.47 | rs753381 | 0.818 |
| rs629301 | *SORT1* | -5.65 | -0.0196 | same direction | LDL | 0.7141 | 0.22 | (A) | (A) |
| rs6511720 | *LDLR* | -6.99 | -0.0318 | same direction | LDL | 0.5239 | 0.11 | (A) | (A) |
| rs7206971 | *OSBPL7* | 0.78 | 0.0457 | same direction | LDL | 0.1569 | 0.49 | rs7214993 | 0.493 |
| rs8017377 | *NYNRIN* | 1.14 | 0.0315 | same direction | LDL | 0.4356 | 0.47 | rs6573766 | 0.118 |
| rs10128711 | *SPTY2D1* | -1.04 | -0.0163 | same direction | TC | 0.6457 | 0.28 | rs11024739 | 1 |
| rs10401969 | *CILP2* | -4.74 | -0.0488 | same direction | TC | 0.6094 | 0.07 | (A) | (A) |
| rs11065987 | *BRAP* | -0.96 | 0.0334 | opposite direction | TC | 0.2933 | 0.42 | rs17696736 | 0.696 |
| rs1169288 | *HNF1A* | 1.42 | 0.045 | same direction | TC | 0.4132 | 0.33 | (A) | (A) |
| rs12027135 | *LDLRAP1* | -1.22 | -0.0305 | same direction | TC | 0.5597 | 0.45 | (A) | (A) |
| rs12916 | *HMGCR* | 2.84 | 0.0003 | same direction | TC | 0.9914 | 0.39 | (A) | (A) |
| rs2000999 | *HPR* | 2.34 | 0.0878 | same direction | TC | 0.02439 | 0.2 | (A) | (A) |
| rs2072183 | *NPC1L1* | 2.01 | -0.0326 | opposite direction | TC | 0.3871 | 0.25 | (A) | (A) |
| rs2081687 | *CYP7A1* | 1.23 | 0.0976 | same direction | TC | 0.003077 | 0.35 | rs8192870 | 0.925 |
| rs2290159 | *RAF1* | -1.42 | -0.0382 | same direction | TC | 0.3466 | 0.22 | rs9817675 | 0.857 |
| rs2902940 | *MAFB* | -1.38 | 0.0111 | opposite direction | TC | 0.7313 | 0.29 | rs6029247 | 0.108 |
| rs3177928 | *HLA* | 2.31 | -0.0547 | opposite direction | TC | 0.2847 | 0.16 | rs17496549 | 0.646 |
| rs492602 | *FLJ36070* | 1.27 | -0.063 | opposite direction | TC | 0.2218 | 0.49 | (A) | (A) |
| rs6882076 | *TIMD4* | -1.98 | 0.0602 | opposite direction | TC | 0.2622 | 0.35 | (A) | (A) |
| rs1042034 |  | -5.99 | -0.0172 | same direction | TG | 0.6511 | 0.22 | (A) | (A) |
| rs11613352 | *LRP1* | -2.7 | -0.045 | same direction | TG | 0.2571 | 0.23 | rs11172134 | 0.802 |
| rs11649653 | *CTF1* | -2.13 | -0.0179 | same direction | TG | 0.5786 | 0.4 | (A) | (A) |
| rs11776767 | *PINX1* | 2.01 | -0.0367 | opposite direction | TG | 0.249 | 0.37 | rs4841317 | 0.125 |
| rs1260326 | *GCKR* | 8.76 | -0.123 | opposite direction | TG | 0.0001345 | 0.41 | (A) | (A) |
| rs12678919 | *LPL* | -13.64 | 0.0592 | opposite direction | TG | 0.5069 | 0.12 | (A) | (A) |
| rs1495741 | *NAT2* | 2.85 | -0.0047 | opposite direction | TG | 0.893 | 0.22 | rs1961456 | 0.524 |
| rs17145738 | *MLXIPL* | -9.32 | 0.0747 | opposite direction | TG | 0.1188 | 0.12 | (A) | (A) |
| rs174546 | *FADS1-2-3* | 3.82 | 0.0526 | same direction | TG | 0.3409 | 0.34 | (A) | (A) |
| rs2068888 | *CYP26A1* | -2.28 | -0.0309 | same direction | TG | 0.3302 | 0.46 | rs4418728 | 1 |
| rs2131925 | *ANGPTL3* | -4.94 | 0.0662 | opposite direction | TG | 0.04468 | 0.32 | rs1748197 | 1 |
| rs2247056 |  | -2.99 | -0.004 | same direction | TG | 0.8998 | 0.25 | rs2523589 | 0.563 |
| rs2929282 | *FRMD5* | 5.13 | -0.0737 | opposite direction | TG | 0.1577 | 0.05 | rs10438303 | 0.237 |
| rs2954029 | *TRIB1* | -5.64 | -0.0283 | same direction | TG | 0.3696 | 0.47 | (A) | (A) |
| rs439401 |  | -5.5 | 0.0116 | opposite direction | TG | 0.7225 | 0.36 | (A) | (A) |
| rs442177 | *KLHL8* | -2.25 | -0.0484 | same direction | TG | 0.1333 | 0.41 | rs3775214 | 0.964 |
| rs5756931 | *PLA2G6* | -1.54 | 0.0932 | opposite direction | TG | 0.004772 | 0.4 | rs4820314 | 0.802 |
| rs645040 | *MSL2L1* | -2.22 | -0.0516 | same direction | TG | 0.3991 | 0.22 | (A) | (A) |
| rs964184 | *APOA1* | 16.95 | 0.0248 | same direction | TG | 0.6875 | 0.13 | rs12286037 | 0.588 |

(A) This was not necessary, as it was directly genotyped

| Supplementary Table 6. Conditional analysis by adjustment of lead SNPs within the *ABCG5/8* region. | | | | | | | | | | | | |
| --- | --- | --- | --- | --- | --- | --- | --- | --- | --- | --- | --- | --- |
| SNP | Position (hg18) | Effect allele | Non-effect allele | Effect allele frequency | Un-conditioned meta-analysis | | Conditoinal analysis 1^a^ | | Conditoinal analysis 2^b^ | | Conditoinal analysis 3^c^ | |
|  |  |  |  |  | Effect | P-value | Effect | P-value | Effect | P-value | Effect | P-value |
| rs4953023 | chr2:43927504 | A | G | 0.066 | 0.734 | 7.41x10^-47^ | - | - | - | - | - | - |
| rs4299376 | chr2:43926080 | T | G | 0.686 | 0.278 | 2.40x10^-18^ | 0.184 | 8.22x10^-8^ | - | - | - | - |
| rs6544718 | chr2:43958429 | T | C | 0.216 | 0.044 | 2.08x10^-14^ | 0.116 | 1.65x10^-3^ | 0.142 | 1.39x10^-4^ | - | - |
| rs6720173 | chr2:43893905 | C | G | 0.159 | 0.262 | 3.81x10^-12^ | 0.165 | 7.46x10^-5^ | 0.154 | 2.15x10^-4^ | 0.151 | 3.09x10^-4^ |
| A total of 77 SNPs were designed in the IBC array, thus we used locus-wide significance level of 0.05/77=6.49x10^-4^ as the cutoff for conditional analysis. In the fourth round of conditional analysis, no marker in the *ABCG5/8* locus had p value < 6.49x10^-4^. So we have four independent signals within this region, i.e. rs4953023 (lead SNP in the un-conditioned meta-analysis), rs4299376 (lead SNP in the first round conditional analysis), rs6544718 (lead SNP in the second round conditional analysis), and rs6720173 (lead SNP in the third round of conditional analysis). ^a^: Conditioning by taking genotypes of rs4953023 as a covariate. ^b^: Genotypes of rs4953023 and rs4299376 as a covariate. ^c^: Genotypes of rs4953023, rs4299376 and rs6544718 as a covariate. | | | | | | | | | | | | |

**Supplementary Figure 1. Comparison between r^2^ values and P values and between r^2^ values and effect sizes for key SNPs associated with GBD**

A) Rs4953023, the locus in *ABCG8* having the greatest effect on GBD.The coding variant rs11887534 (D19H) had been identified to be associated with cholesterol gallbladder stones in previous GWAS and linkage studies [1, 2], but rs11887534 failed the design and is not on the IBC chip. Rs4953023, which is located in intron 3 of *ABCG8*, had the largest effect size and strongest statistical evidence, (p=3.94x10^-50^, effect size=0.7027). This SNP is in perfect LD with D19H (r^2^=1 in Europeans in HapMap). Because of this perfect LD, we confirm D19H of *ABCG8* as a genetic risk factor for gallstones. Another SNP in *ABCG8* (rs4299376), was found to have a significant effect on GBD (p=9.17x10^-22^) but had a smaller effect size of 0.2877. The r^2^ between rs4299376 and C54Y, which is a reported coding SNP for gallstone disease in Taiwanese, was 0.209 [3]. We hypothesize that these two SNPs may be in one linkage disequilibrium block and may represent the same functional mechanism or (unknown) causal SNP.


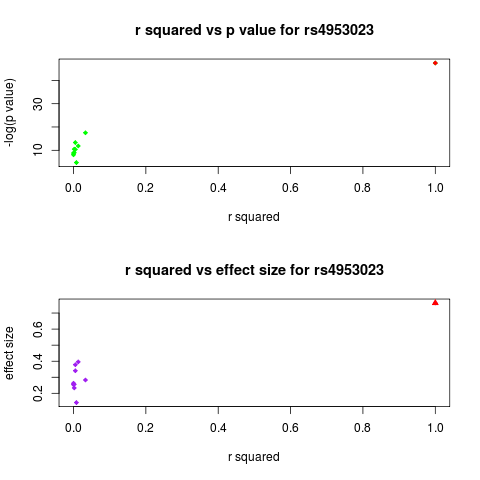


B) Rs10439467 in *ABCG5*, which is located in intron 10 in gene *ABCG5*, was the fifth most significantly associated SNP (p=1.54x10^-14^, effect size=0.3889) in this meta-analysis. It appears to mark a novel independent effect which does not appear to have been well represented in earlier chip-based analyses. This SNP is essentially in linkage equilibrium with E604Q, which is the only previously reported coding SNP for gallstones in *ABCG5* (r^2^ = 0.095 in Europeans in HapMap) [2, 3]. This confirmed its independence of effect on GBD. Four other significant SNPs in the *ABCG5* region were no longer retained as independent predictors, respectively rs4953019 (p=5.81x10^-13^, effect size=0.3382, r^2^=0.352), rs10208987 (p=3.17x10^-17^, effect size=-0.3838, r^2^=0.352), rs10201851 (p=5.03x10^-11^, effect size=-0.2610, r^2^=0.196) and rs4148189 (p=1.02x10^-9^, effect size=0.2461, r^2^=0.196).


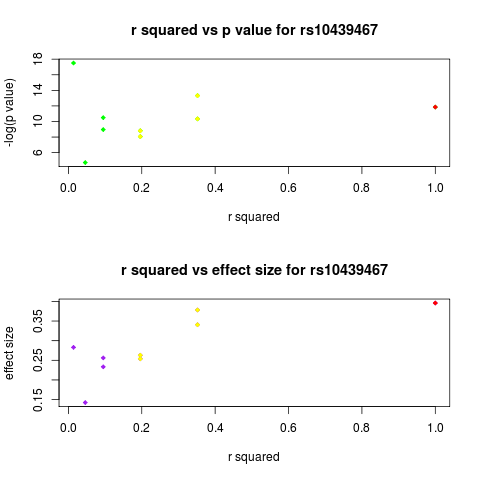


C) rs6720173 (E604Q) is the only reported coding SNP showing a significant association in our study (p=1.66x10^-14^, effect size= 0.2697) [2, 3]. Two significant SNPs rs2278357 (r^2^=1, p=1.82x10^-12^, effect size=0.2463) and rs4245786 (r^2^=0.234, p=1.88x10^-6^, effect size= -0.1454) are in LD with E604Q.


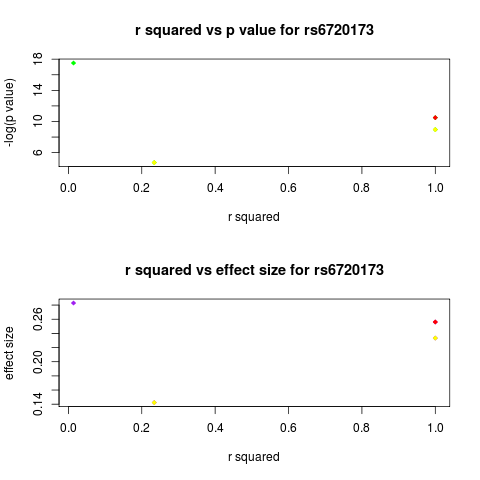


**Supplementary Figure 2.** A core network of MESH-defined gall bladder disease genes that interact directly with associated gene loci reported in this study.

Genes reported in this study are indicated by a red dot. All 142 genes constituting the original MESH-defined network are listed in supplementary table Y. The custom network was prepared using GeneGO metacore (Thomson Reuters).


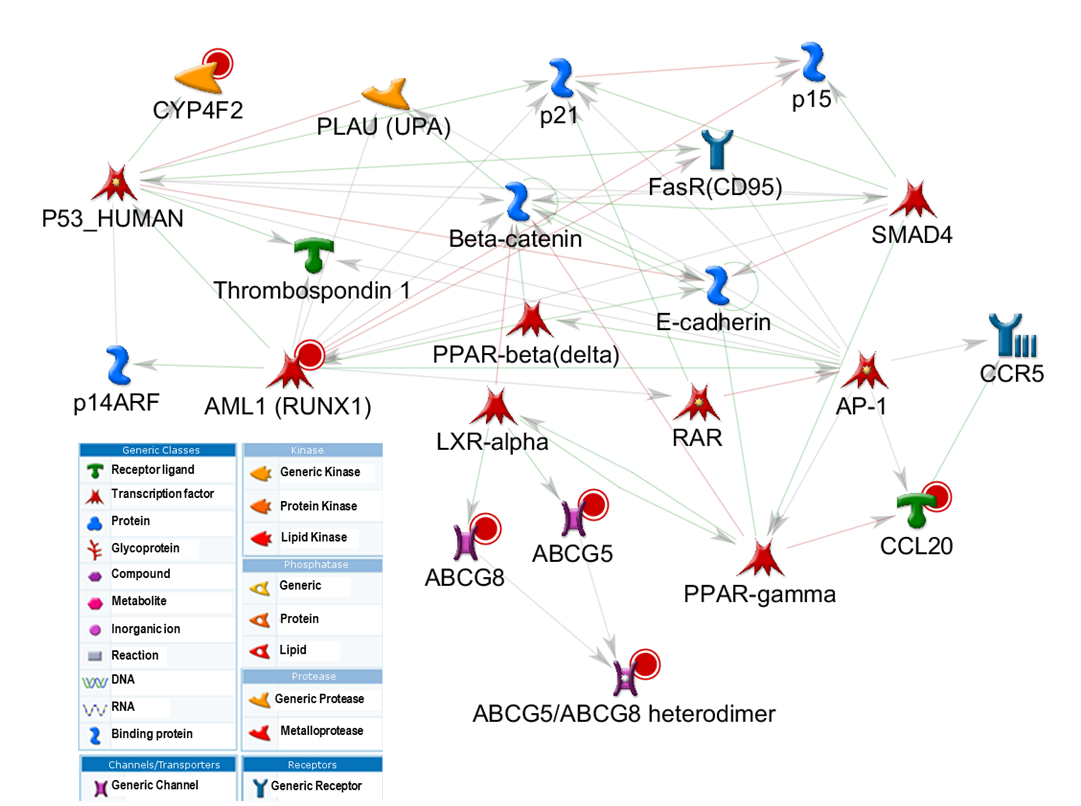


**Supplementary Figure 3.** An extended MESH-defined gall bladder disease gene network including interacting genes with associations reported in this study. Genes reported in this study are indicated by a red dot. All 142 genes constituting the network are listed in supplementary table Y. The custom network was prepared using GeneGO metacore (Thomson Reuters).
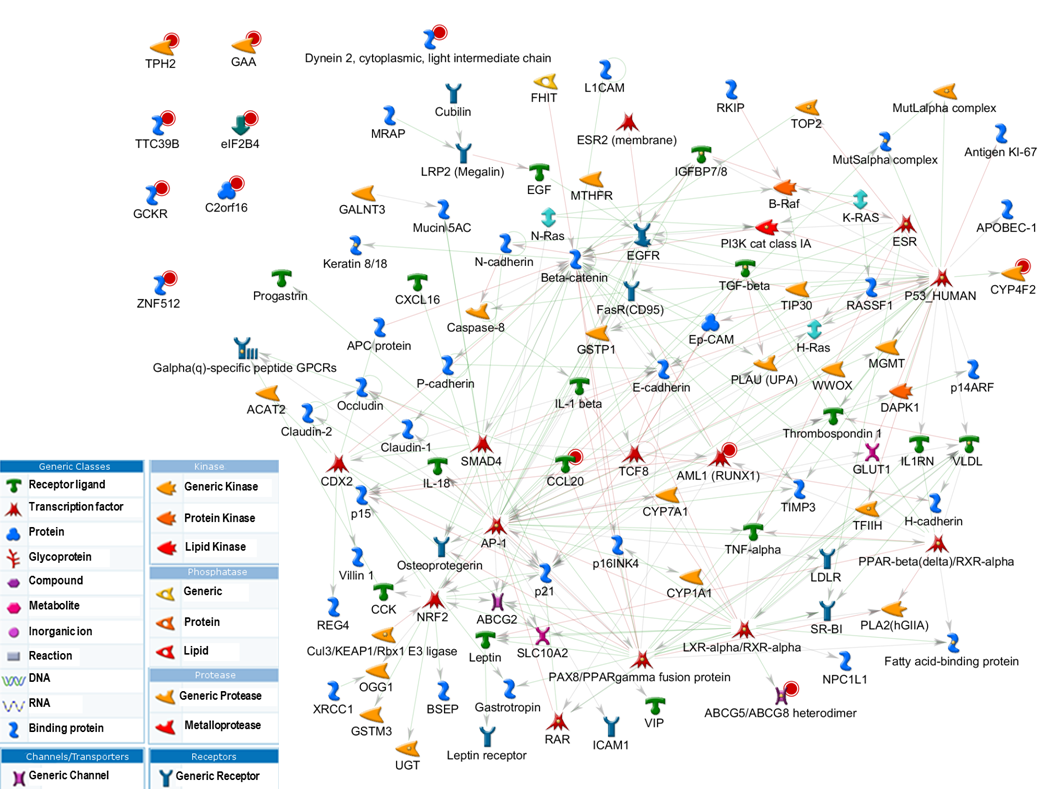


**Supplementary figures 4-15**
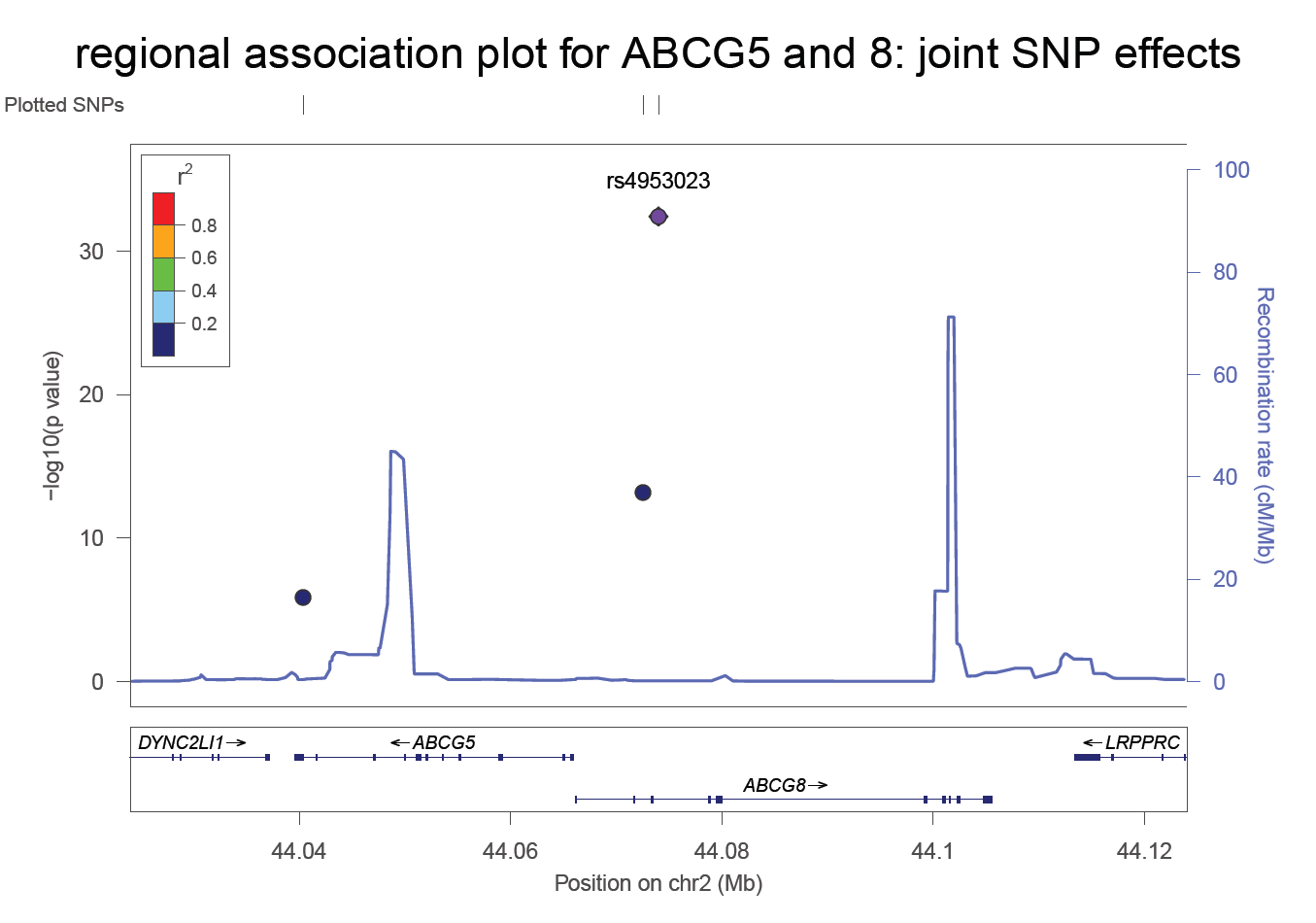


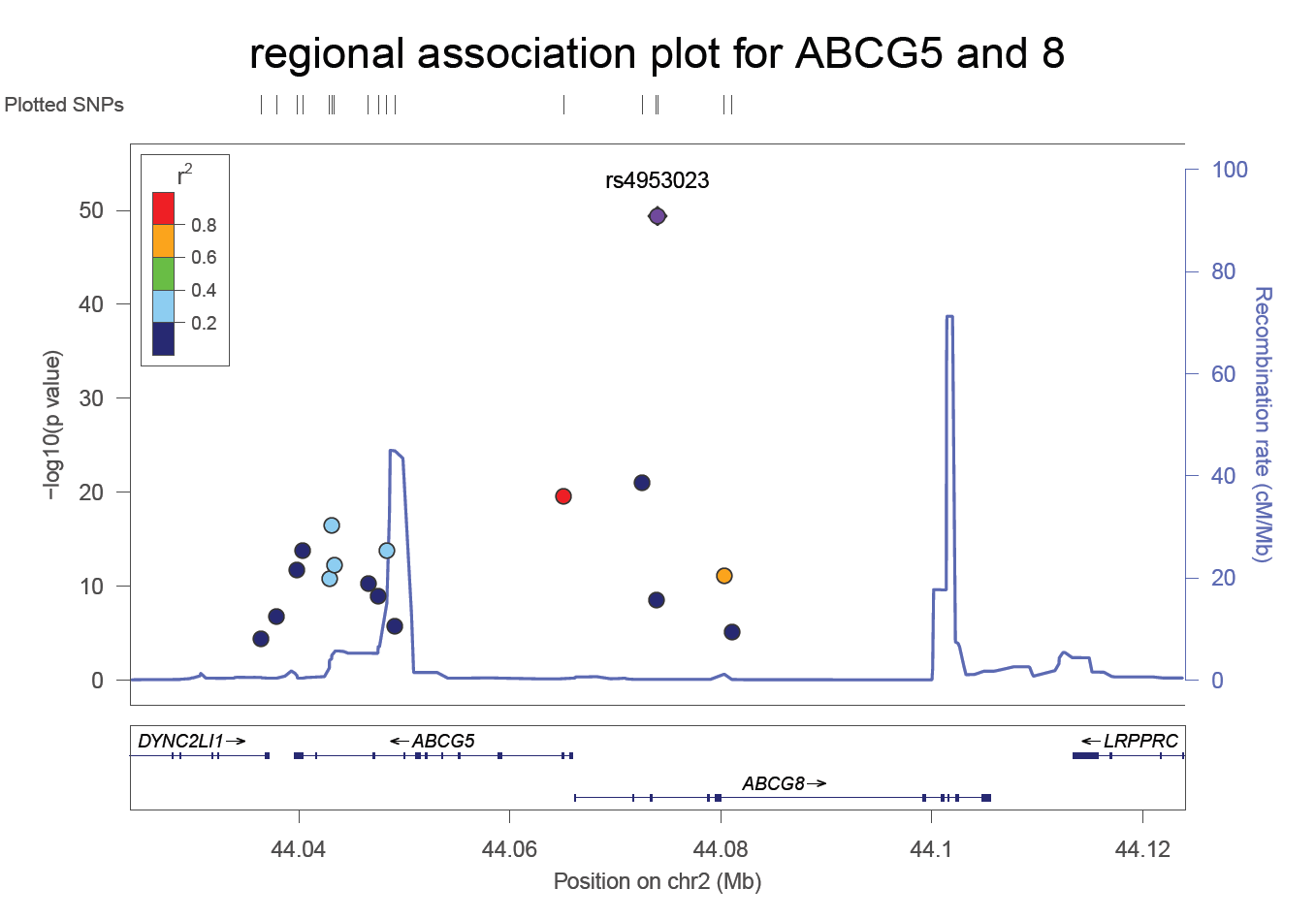


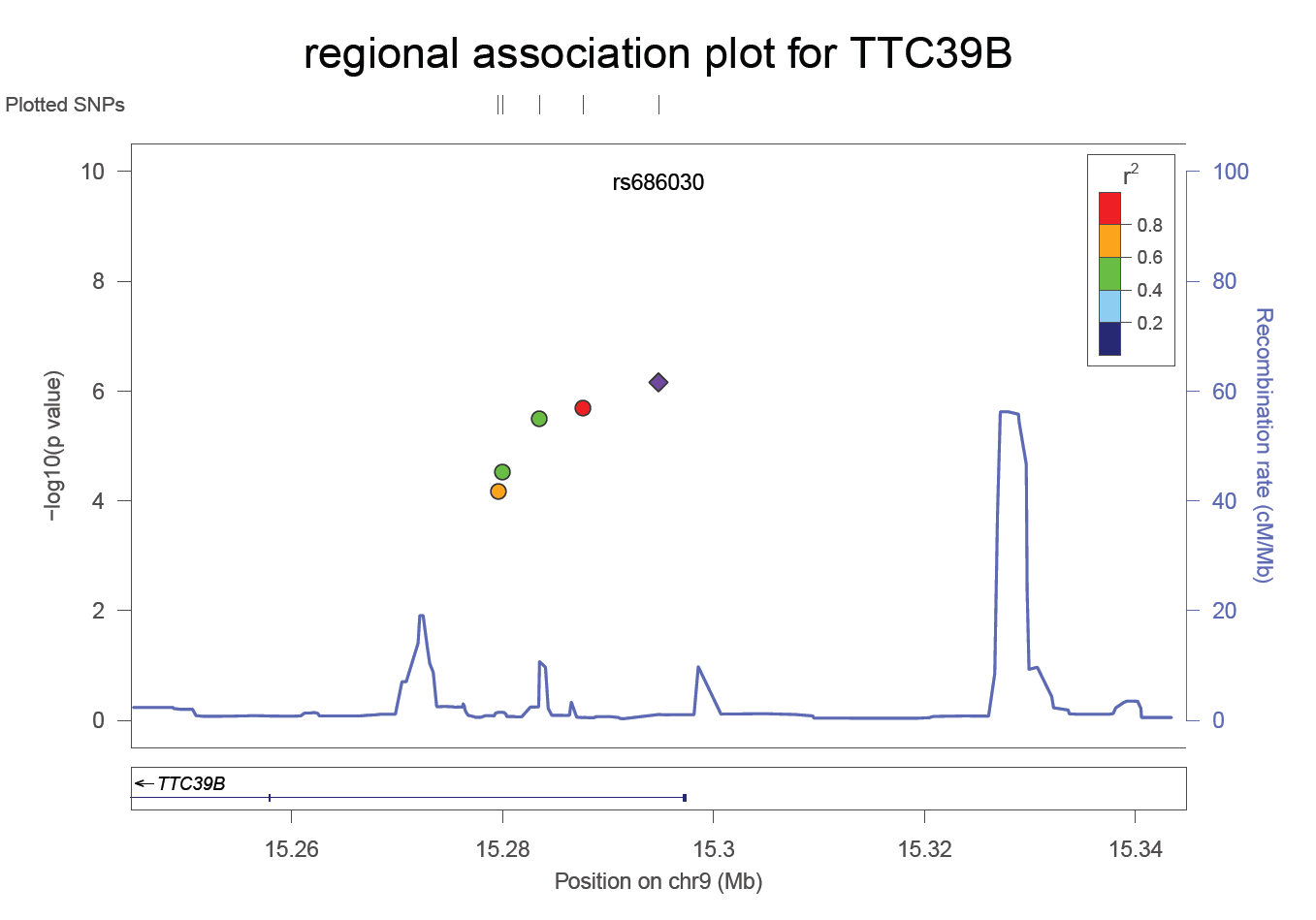


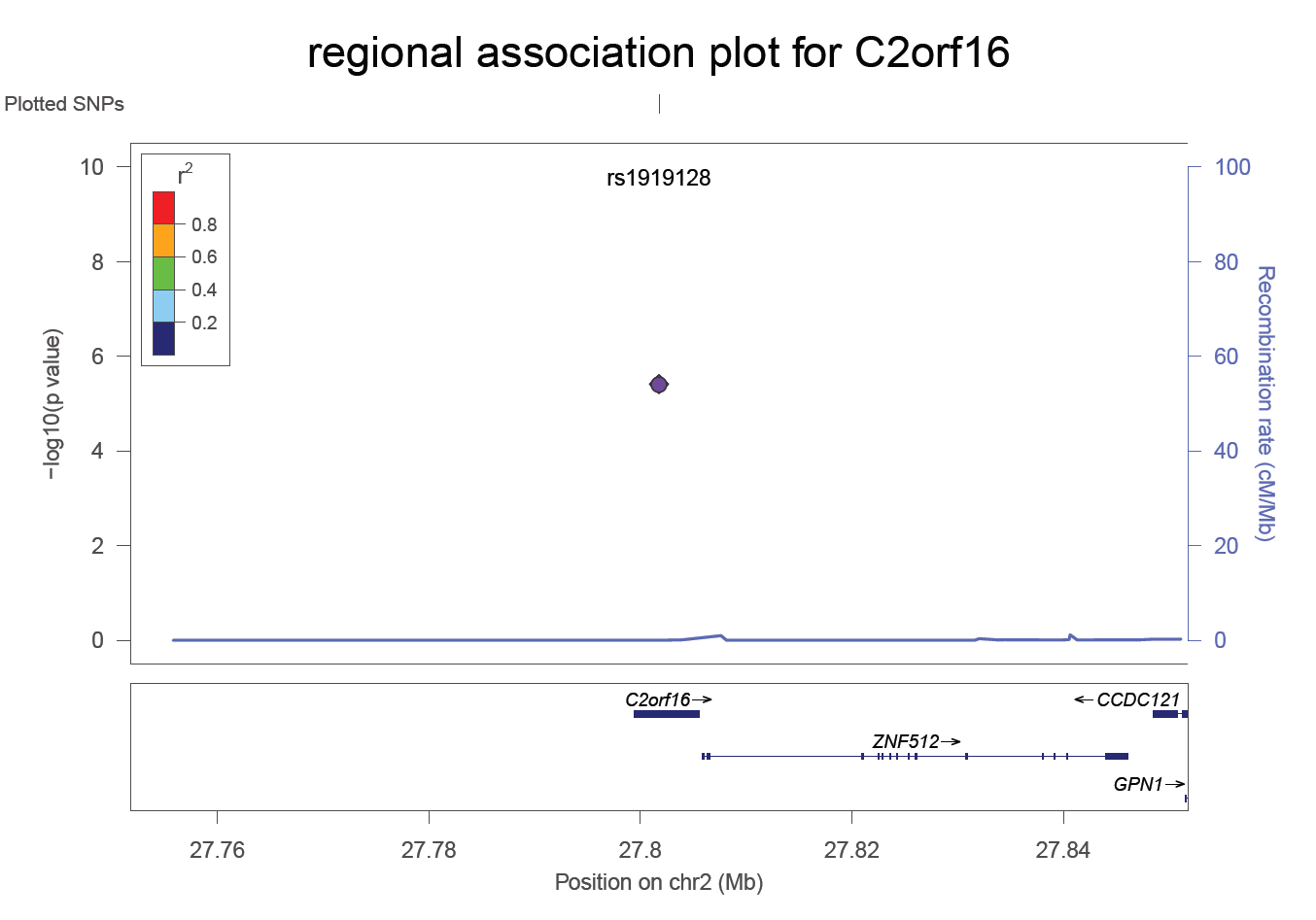


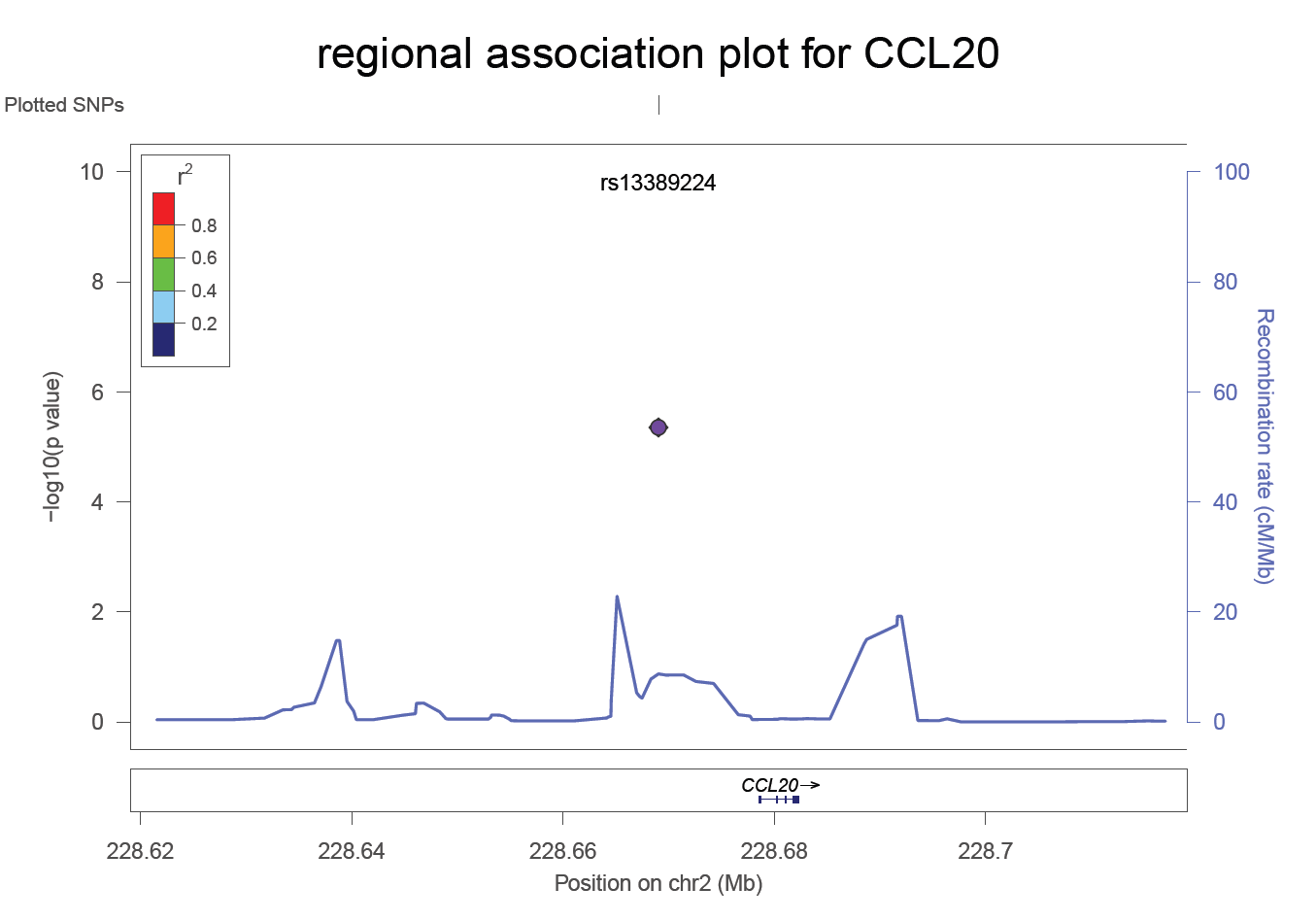


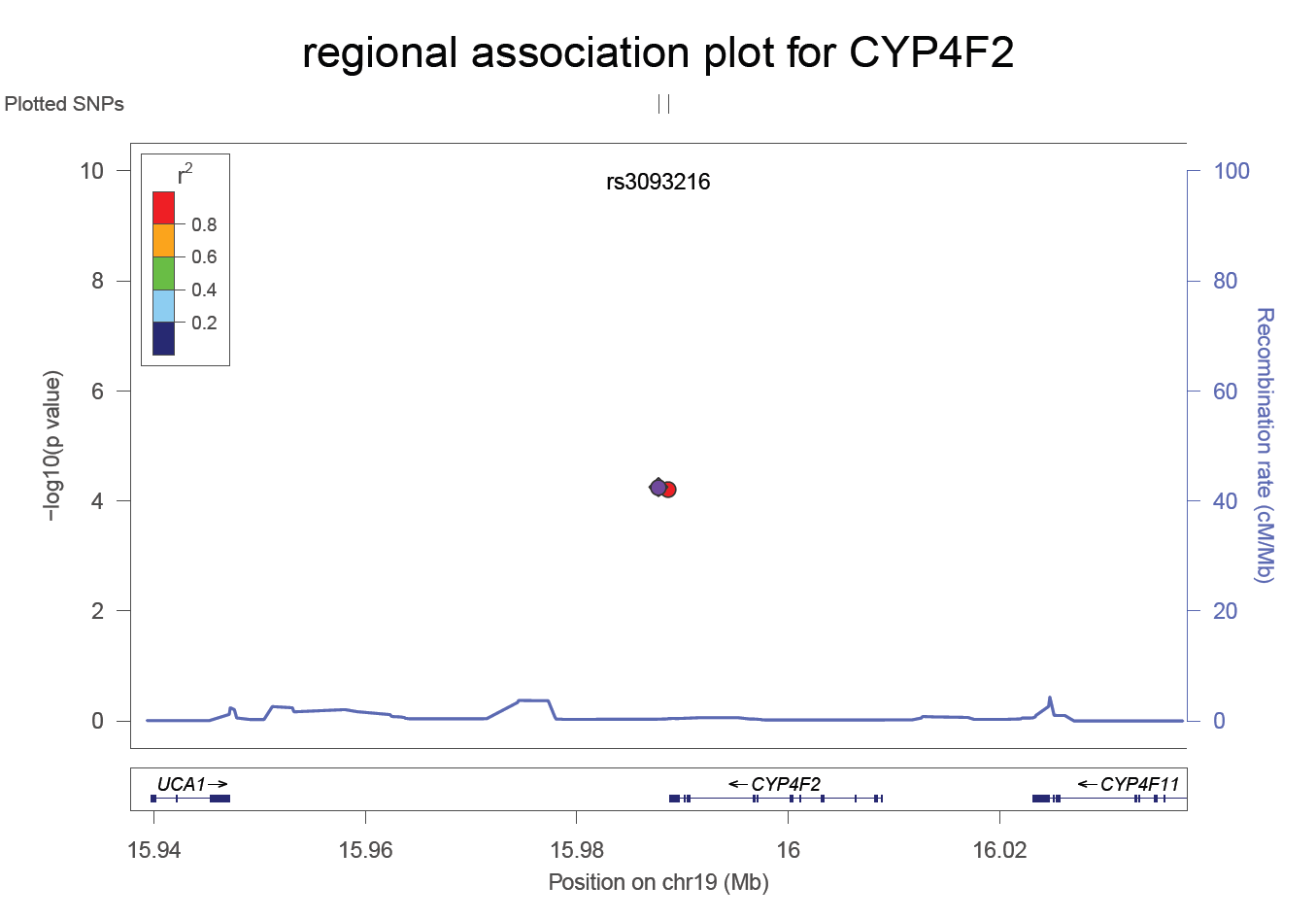


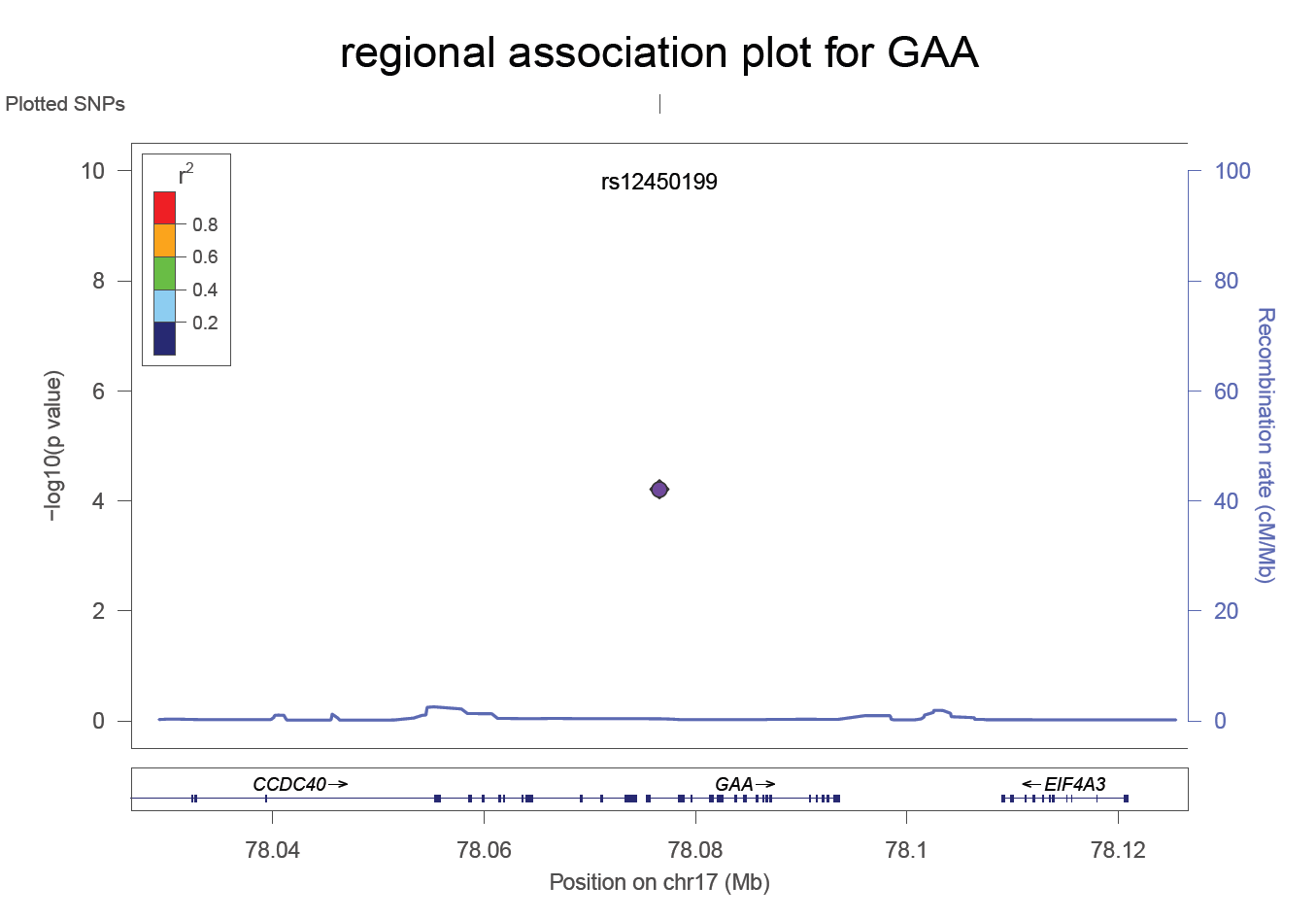


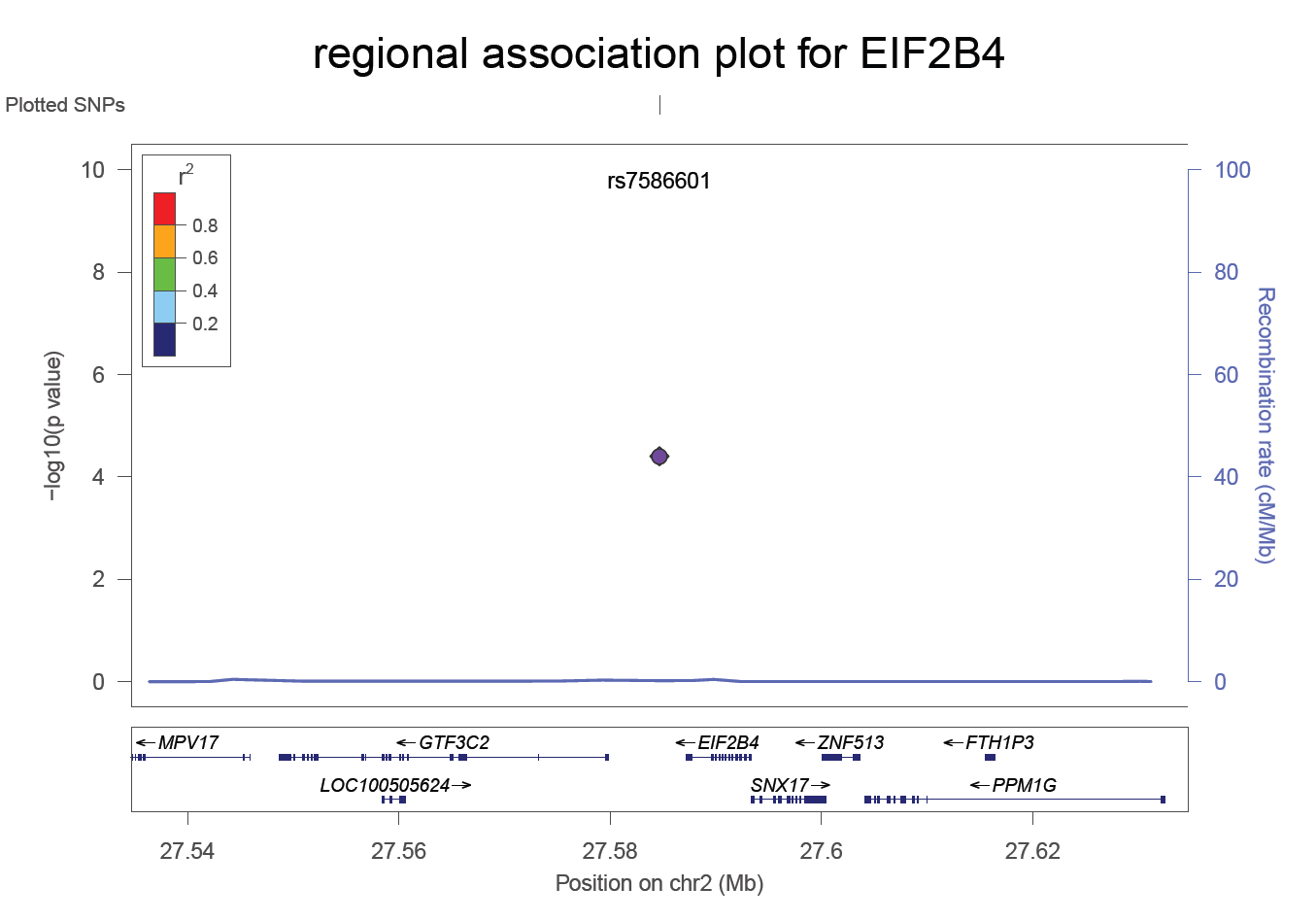


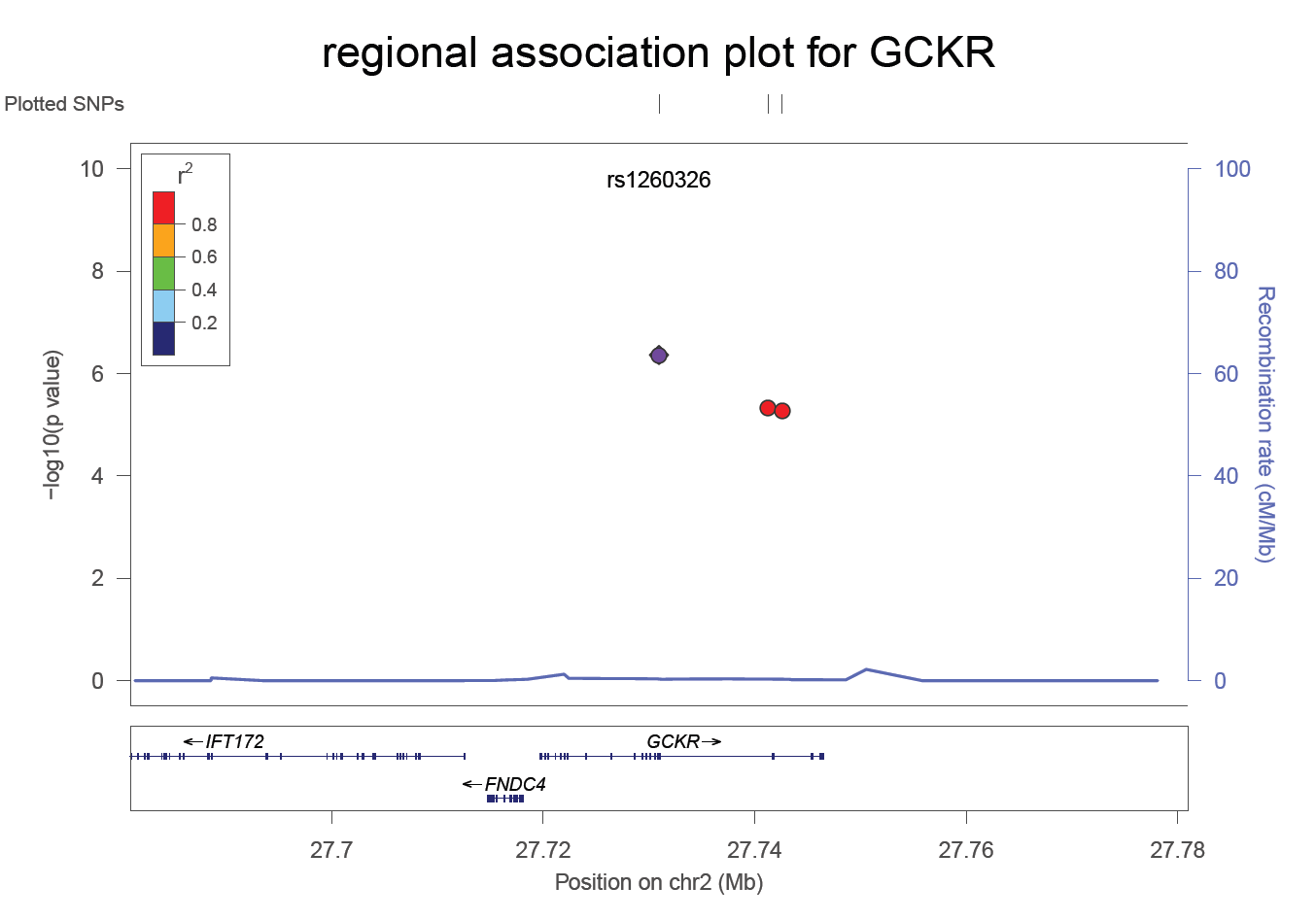


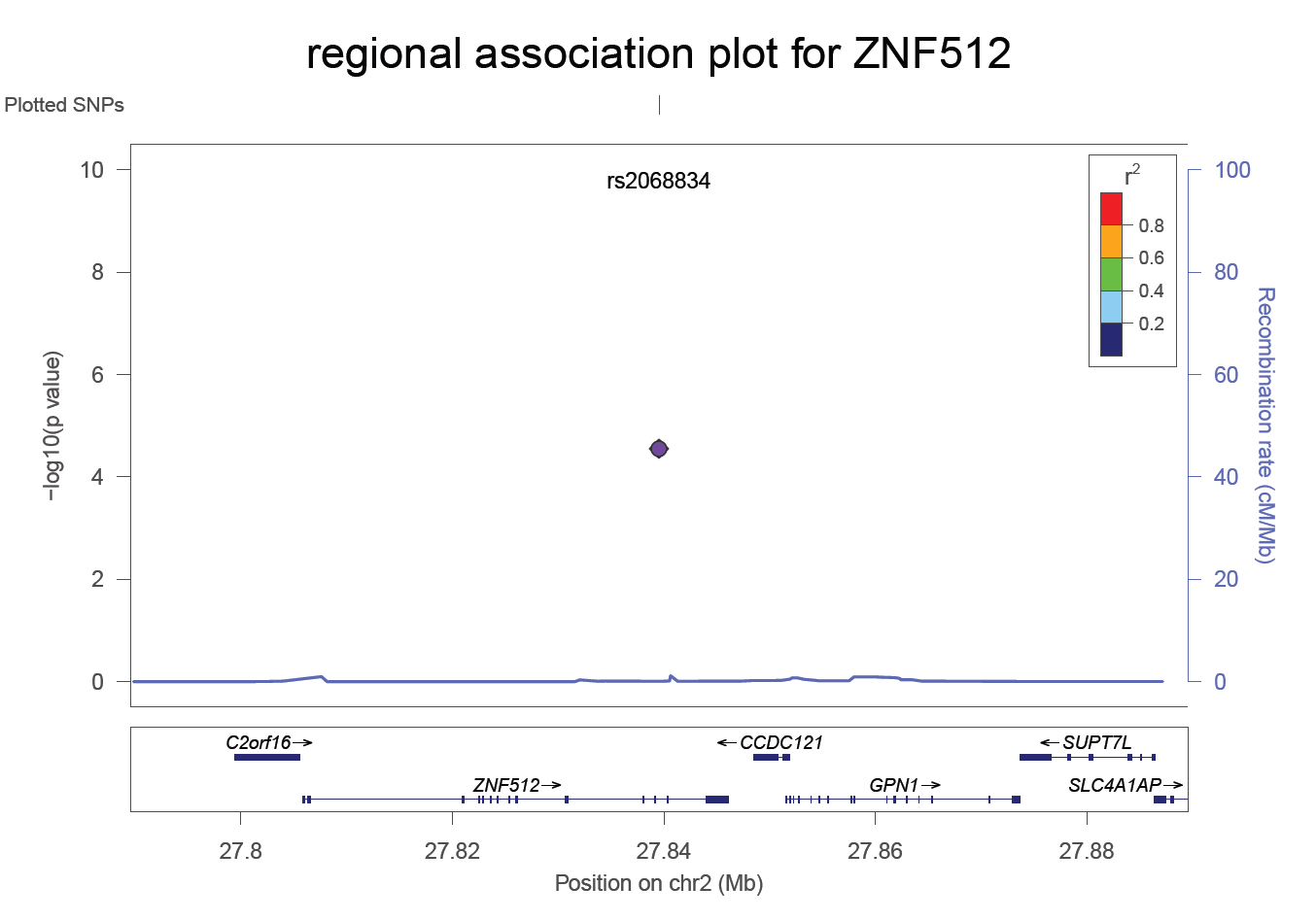


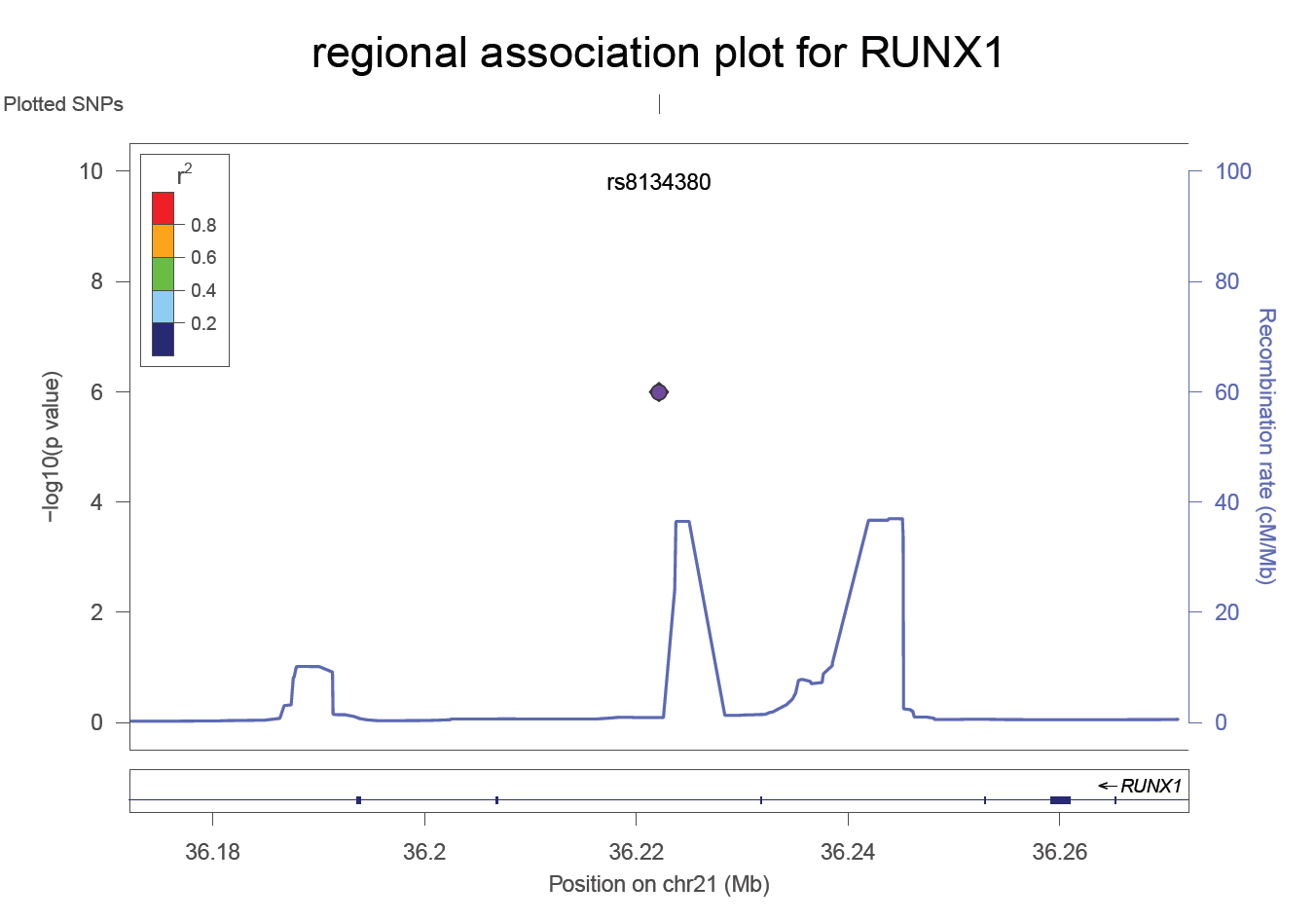


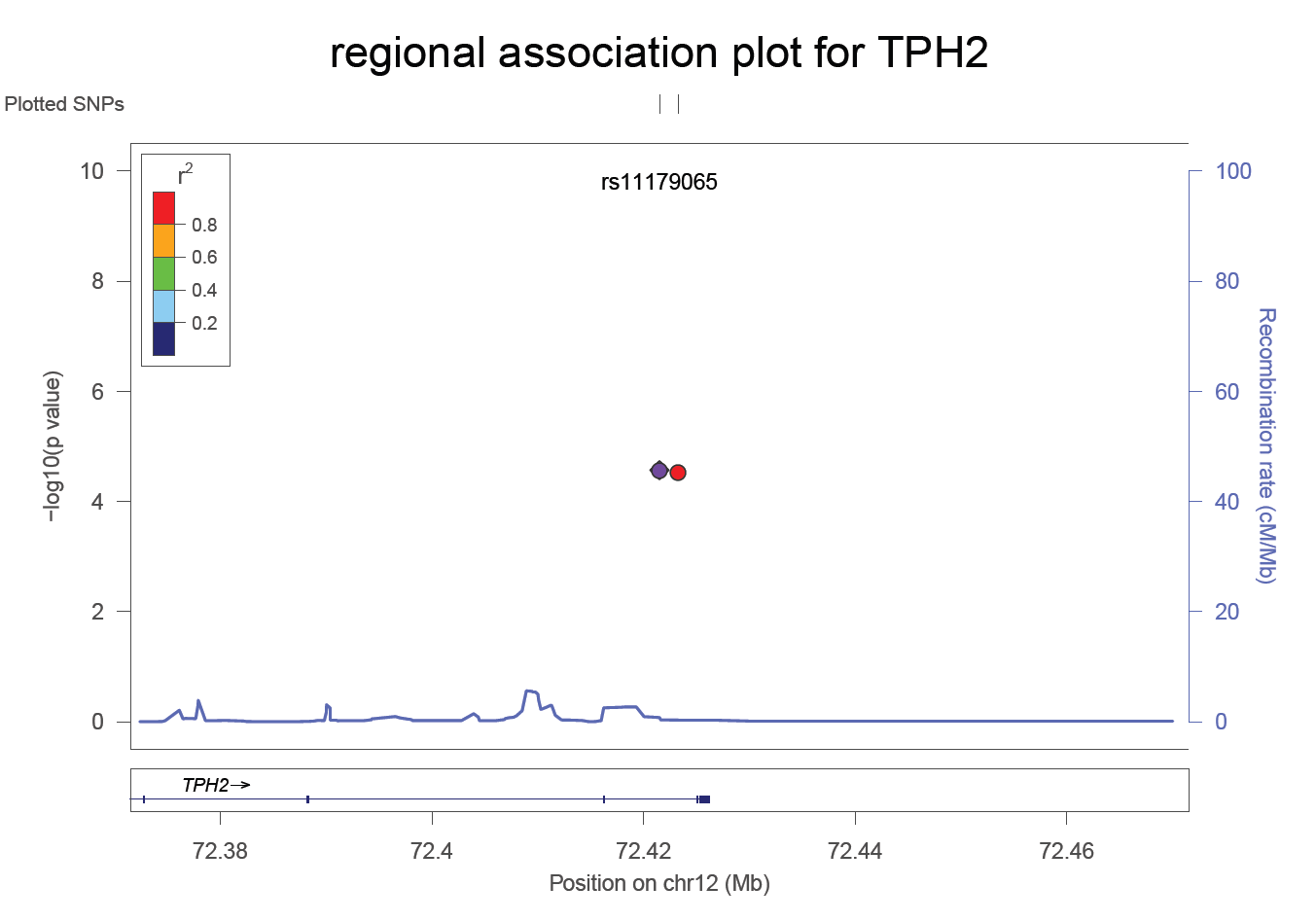

Supplement: Supplementary Information [file ejhg201563x1.doc]
